# Traditional Chinese medicine–based modulation of gut microbiota in coronary heart disease: probiotic synergy and translational perspectives

**DOI:** 10.3389/fcvm.2026.1857702

**Published:** 2026-06-02

**Authors:** Hong Xu, Jingqing Hu

**Affiliations:** 1 Chengdu University of Traditional Chinese Medicine, Chengdu, China; 2 Tianjin University of Traditional Chinese Medicine, TianJin, China

**Keywords:** coronary heart disease (CHD), gut microbiota, probiotics, SCFAs, traditional Chinese medicine (TCM), trimethylamine N-Oxide (TMAO)

## Abstract

Gut microbiota has emerged as an important contributor to the pathogenesis and progression of coronary heart disease (CHD). Increasing evidence indicates that gut dysbiosis promotes atherosclerosis and cardiovascular dysfunction through microbiota-derived metabolites, including trimethylamine N-oxide (TMAO), short-chain fatty acids (SCFAs), and bile acid-related pathways, as well as through effects on inflammation, cholesterol metabolism, endothelial injury, and intestinal barrier integrity. In recent years, phytomedicine, particularly Traditional Chinese Medicine (TCM), has attracted growing attention as a microbiota-modulating strategy because of its multi-component, multi-target, and system-level regulatory properties. In parallel, probiotics may provide targeted supplementation of beneficial strains and functional pathways. This review summarizes the major microbiota-mediated mechanisms involved in CHD and examines how phytomedicine regulates gut microbial composition, microbial metabolism, host inflammatory responses, and barrier function. We further discuss the complementary potential of phytomedicine and probiotics, highlighting their possible synergistic roles in restoring microbial ecology and improving cardiometabolic homeostasis. In addition, we critically evaluate current limitations in the field, including insufficient standardization of phytomedicine–probiotic combinations, heterogeneity of host–microbiota responses, limited clinical evidence, and unresolved long-term safety issues. Overall, this review provides an ethnopharmacology-oriented and mechanistically integrated perspective on microbiota-targeted interventions in CHD, and suggests that phytomedicine-based modulation, alone or in combination with probiotics, may represent a promising direction for future precision prevention and management of CHD.

## Graphical Abstract

**Figure:**
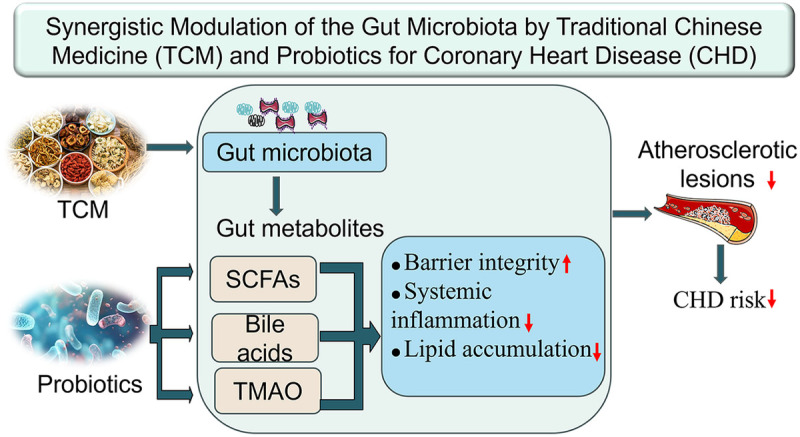

## Highlights

- Gut microbiota is closely involved in the progression of CHD.
- Phytomedicine modulates microbial metabolism, inflammation, and intestinal barrier function.
- TMAO, SCFAs, and bile acid pathways link gut dysbiosis to CHD pathology.
- Probiotics may complement phytomedicine through targeted microbial functional support.
- Standardization and translational validation remain key challenges for clinical application.

## 1 Introduction

Coronary heart disease (CHD) is one of the leading causes of death worldwide, and its disease burden is still on the rise despite advances in drug and interventional therapy (1, 2). This trend is largely driven by population aging, high-fat diets, sedentary lifestyles, and the increasing prevalence of metabolic syndrome in younger populations (2). Modeling studies predict that CHD incidence and mortality will continue to increase globally by 2050, particularly in developing countries (1). Although thrombolysis, percutaneous coronary intervention, and guideline-based secondary prevention medications have markedly reduced acute-phase mortality, long-term follow-up studies demonstrate that cardiovascular event recurrence remains substantial. For instance, among acute coronary syndrome survivors undergoing percutaneous coronary intervention, recurrent myocardial infarction, stroke, or cardiac death remain common during follow-up (3–5). These observations indicate that current therapeutic strategies do not fully prevent disease progression, prompting the search for novel pathogenic mechanisms and interventional targets.

Traditional risk factors (such as dyslipidemia, glucose metabolism disorder, and chronic inflammation) are closely related to the state of gut microbiota, which makes “microbiota-metabolism-immunity” an important entry point for explaining the complex pathology of CHD (6, 7). In particular, microbial metabolites such as trimethylamine-N-oxide (TMAO) secondary bile acids, and short-chain fatty acids (SCFAs) can cross the intestinal barrier to affect the systemic metabolic environment, thereby promoting atherosclerosis and vascular dysfunction (1–3, 5, 8–11).

Emerging evidence supports a microbiota–metabolism–immunity–vascular network underlying atherosclerosis progression. Accumulating evidence indicates that patients with CHD generally have detectable microbiota characteristics: the production of SCFAs is reduced, the proportion of TMAO-producing microbiota is increased, and the endotoxin load associated with Gram-negative bacteria is increased (1, 6, 7, 12). These features may also serve as early indicators of cardiovascular risk. Recent genetic evidence reinforces the potential causal role of gut microbiota in CHD. A two-sample Mendelian randomization study published in 2024 demonstrated that genetically predicted abundances of certain gut microbial taxa—including *Actinobacteria* (class), *Bifidobacterium*, *Butyricicoccus*, *Oxalobacter* and *Turicibacter* (genera)—were suggestively associated with increased risk of CHD (6). This finding lends causal-inference support to the gut–heart axis hypothesis, highlighting gut microbiota as a candidate for preventive or therapeutic targeting in CHD (6). TMAO is associated with increased cardiovascular risk, whereas SCFAs exhibit protective effects on metabolism, barrier integrity, and inflammation (3, 5, 8–11, 13, 14). The dynamic balance between the two may largely determine the pathological direction of patients with CHD.

It is worth emphasizing that the microbiota does not determine the disease alone, but promotes the progression of the disease under the interaction of genetic background, dietary patterns and environmental factors. For example, long-term intake of high-choline diet will amplify the metabolic potential of TMAO-producing bacteria (14); while high-fiber diet can improve metabolic status by increasing SCFAs level (15). This interaction highlights the microbiota as a modifiable intervention target rather than a passive disease marker.

Both Traditional Chinese Medicine (TCM) and probiotics have shown considerable potential in regulating the composition of the microbiota and the metabolic network, but the focus of the two is not the same. TCM compound usually improves intestinal barrier, inflammatory response and metabolic disorder through the comprehensive effect of multiple components, and its regulation range is wider (16–24); probiotics can play an intervention role in bile acid, cholesterol absorption and inflammation regulation by supplementing specific strains (25–31). The two have their own advantages, which provide a new direction for the development of “compound microecological therapy”. Combining the two into the same intervention system may bring more stable and consistent metabolic benefits.

However, there are still some unresolved problems. For example, there is no unified standard for strain selection, dosage, course of treatment, and compatibility principles with TCM. Although many mechanisms are supported by animal or *in vitro* evidence, the causal chain at the population level is not complete.

In addition, substantial interindividual variability in gut microbiota composition continues to pose challenges for the development of precise intervention strategies (32–34). Accordingly, this review reorganizes current evidence and evaluates the synergistic potential of TCM and probiotics from both mechanistic and clinical perspectives.

This review aims to: (1) summarize mechanisms linking microbiota and CHD; (2) outline independent and synergistic pathways of TCM and probiotics; (3) evaluate current evidence quality and translational potential; (4) identify future research priorities, including standardization, individualized strategies, and AI-assisted prediction. This interdisciplinary synthesis provides a systematic theoretical basis for microbiota-based precision regulation strategies in CHD. How to transform these basic understandings into feasible, controllable, and repeatable clinical interventions in the future will be the key to promoting the upgrading of CHD prevention and treatment strategies.

## 2 Methods: literature search and study selection

A structured literature search was conducted in PubMed and Web of Science from database inception to March 2026 to identify studies examining the interactions among CHD, gut microbiota, TCM, and probiotics. The search strategy combined Medical Subject Headings (MeSH) and free-text terms, including (“coronary heart disease” OR “CHD”), (“gut microbiota” OR “intestinal microbiota” OR “microbiome”), (“traditional Chinese medicine” OR “TCM” OR “herbal medicine”), and (“probiotics”), with appropriate adaptation for each database. This review was conducted in accordance with narrative review principles with elements of systematic reporting to enhance transparency.

Eligible studies included original research articles (*in vivo*, *in vitro*, and clinical studies) and high-quality reviews providing relevant mechanistic or clinical evidence. Conference abstracts, editorials, duplicate publications, and studies lacking sufficient methodological detail or outcome data were excluded. Study selection was performed through title and abstract screening followed by full-text assessment. Reference lists of included studies were also screened to identify additional relevant publications.

## 3 Gut microbiota dysbiosis is a novel risk factor for CHD

With the popularization of metagenomics, metabolomics and microecology sequencing technology, the relationship between gut microbiota and cardiovascular diseases has gradually shifted from “correlation speculation” to an evidence system with mechanism support. Gut microbiota is not merely a passive participant in digestion,but can have a profound impact on the occurrence and progression of CHD by affecting multiple pathways such as immune regulation, energy metabolism, oxidative stress and inflammatory response (35). Therefore, a new understanding is forming: dysbiosis may be an active driving factor in the initiation of atherosclerosis, rather than a secondary phenomenon (36). The gut microbiota is likely to be a key “transmission structure” between metabolic disorders and vascular damage (32).

### 3.1 Evidence linking gut microbiota and CHD

Although a unified theoretical model across fields has not yet been formed, current evidence consistently supports a multi-level pathological association between gut microbiota and CHD. Systematic reviews have also pointed out that dysbiosis participates in metabolic disorders, inflammatory response activation and intestinal barrier damage, which are the pathological core of atherosclerosis. Zhang et al. (1) further abstracted this association into the “gut-heart axis”, emphasizing the continuous impact of the flora on blood pressure regulation, endothelial vasodilation and contraction response, inflammation level and other metabolic processes (7).

On the other hand, metabolomics studies further reinforce this link. Gut microbiota-derived metabolites, such as TMAO, SCFAs, aromatic metabolites, and secondary bile acids, are associated with cardiovascular risk (2). When SCFA-producing bacteria decrease and TMAO-associated bacteria increase, cardiovascular risk correspondingly rises (1). Clinical studies have also revealed this microecological feature. Patients with CHD generally had a significant reduction in butyrate-producing bacteria, while some genera highly associated with cardiovascular events had a stable pattern of continuous increase (37–39). Based on these observations, researchers have proposed the “gut-intestine-vascular axis” framework (40).

These multi-dimensional data together support a more solid conclusion: the gut microbiota plays the role of “active participant” rather than “bystander” in CHD.

### 3.2 The potential of the gut microbiota as an intervention target

In recent years, research on gut microbiota and CHD has increased rapidly. A bibliometric analysis of 457 articles (2002–2022) showed that the number of related publications has increased significantly since 2014, reaching a peak in 2019–2022, and the research hotspots have gradually focused on the association between inflammation, metabolism (including TMAO, SCFAs, bile acids), diet/nutrition, probiotics, etc. and CHD (12). The gut microbiota is shifting from a “risk marker” to an “intervention target.” Strategies under investigation include dietary modulation, probiotics/prebiotics, TMAO-targeting small molecules, and fecal microbiota transplantation, all showing varying degrees of improvement in metabolic and inflammatory parameters (2).

Compared with drugs with fixed targets, the microbiota represents a dynamic ecosystem that can be reshaped by diet, drugs, and environment factors. A key unresolved issue remains the development of controllable, standardized, and individualized microbiota-targeted strategies, which will depend on advances in clinical trials and multi-omics research.

### 3.3 Theoretical basis and research gap of synergistic intervention of probiotics and TCM

TCM has gradually shown its unique advantages in the study of intestinal microecology due to its multi-component, multi-target, and multi-level biological activity. For example, the classic prescriptions such as Simiao Yong'an Decoction and Shengmai Drink not only have the effects of strengthening the spleen, replenishing qi and promoting blood circulation in the theory of TCM, but also show the value of improving the intestinal barrier and restoring the balance of flora in modern research (41, 42). Further studies have confirmed that the polysaccharides, flavonoids, alkaloids and other components in TCM have selective effects on promoting the proliferation of probiotics or inhibiting pathogenic bacteria (36).

Probiotics provide a more targeted and precise way to supplement. *Lactobacillus*, *Bifidobacterium*, and strains with the ability to produce SCFAs can directly supplement key metabolic functions and improve inflammatory states and metabolic disorders (43, 44). The combined intervention of TCM and probiotics has significant synergistic advantages: TCM improves the intestinal environment and provides a more stable matrix for probiotic colonization; probiotics accelerate the biotransformation of active ingredients of TCM and increase the yield of active metabolites (45). However, three gaps remain: ①lack of standardized TCM–probiotic compatibility systems (46); ②unclear functional genes and signaling pathways underlying synergy (47); ③absence of individualized strategies based on microbiome characteristics (48).

## 4 Association mechanisms linking CHD and gut microbiota dysbiosis

**Figure 1:**
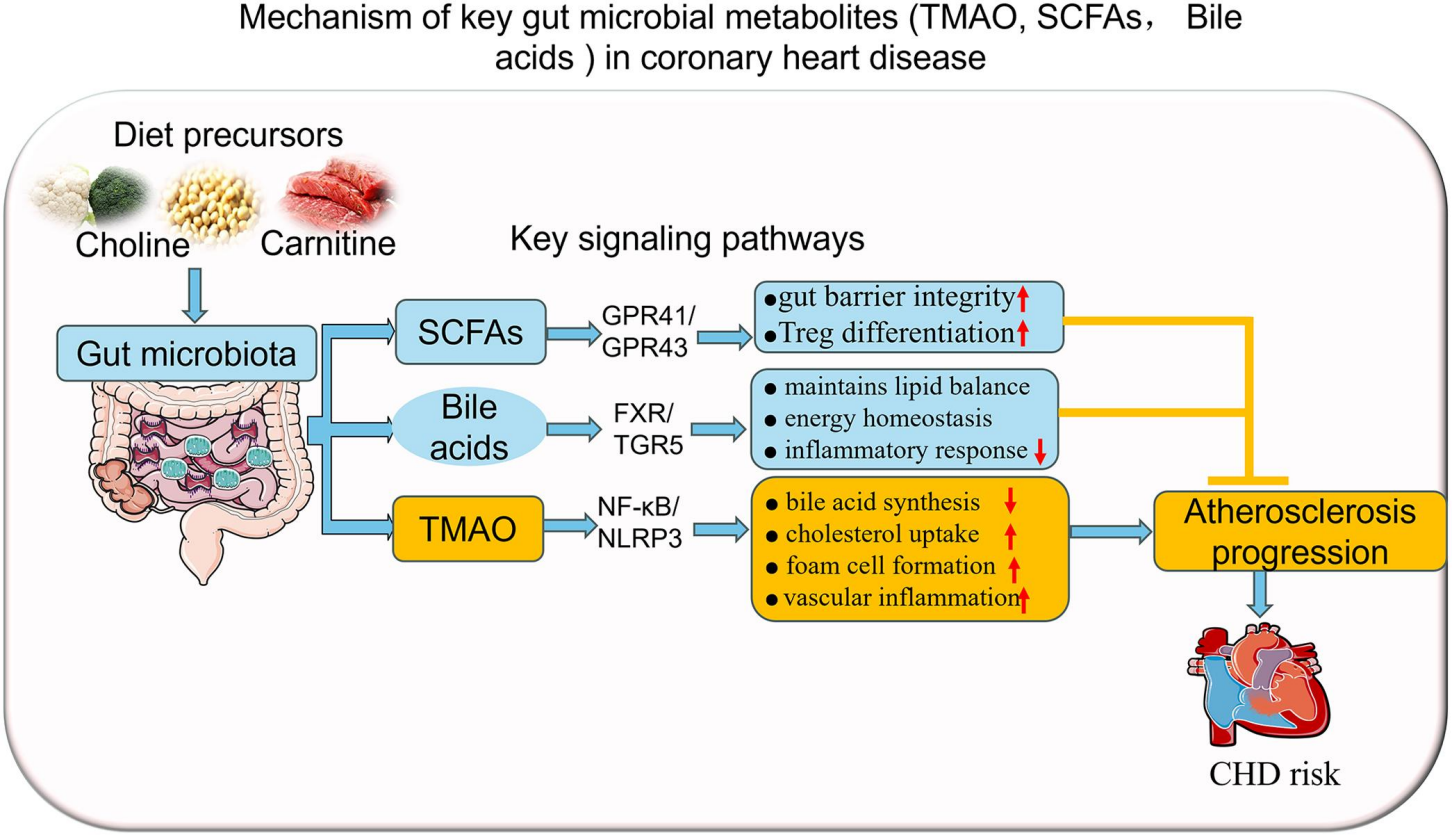
Key gut microbial metabolites and their pathogenic mechanisms in CHD. Dietary precursors such as choline, carnitine, and dietary fiber are metabolized by the gut microbiota into TMAO, SCFAs, and secondary bile acids. These metabolites regulate inflammation, lipid metabolism, intestinal barrier integrity, and endothelial function via NF-*κ*B, NLRP3, GPR41/43, FXR, and TGR5 pathways, thereby influencing atherosclerosis progression and CHD risk.

Current evidence supports a multi-layer association between gut microbiota dysbiosis and CHD, involving altered microbial metabolites, structural imbalance, barrier dysfunction, immune-inflammatory activation, and host metabolic disturbance (Figure 1 and Table 1).

**Table 1:** Key gut microbial metabolites, signaling pathways, and their roles in CHD.

Metabolite	Major sources/precursors	Key receptors/pathways	Main mechanisms of action	Effects on CHD	References
TMAO	Gut microbial metabolism of dietary choline, carnitine, betaine	NF-*κ*B, NLRP3 inflammasome, endothelial dysfunction pathways	Inhibits bile acid synthesis; promotes macrophage cholesterol uptake and foam cell formation; enhances platelet activation and vascular inflammation	Promotes atherosclerosis progression and increases CHD risk	(3, 5, 8, 9)
SCFAs	Microbial fermentation of dietary fibers (acetate, propionate, butyrate)	GPR41, GPR43, HDAC inhibition	Enhances gut barrier integrity; induces Treg differentiation; suppresses inflammatory cytokines; improves insulin sensitivity	Exerts anti-inflammatory, anti-atherogenic, and cardioprotective effects	(6, 10)
Secondary Bile Acids	Microbial conversion of primary bile acids	FXR, TGR5 signaling pathways	Regulates lipid and cholesterol metabolism; modulates energy homeostasis and inflammatory response	Maintains lipid balance and reduces vascular inflammation	(11)

### 4.1 TMAO and SCFAs: two major metabolite axes in atherosclerosis

TMAO and SCFAs represent two key microbial metabolite pathways with opposing effects. TMAO has been linked to impaired bile acid metabolism, foam cell formation, vascular inflammation, and platelet activation, thereby promoting atherosclerosis (3, 8, 49). Clinical and prospective studies suggest that elevated TMAO is associated with plaque instability and increased cardiovascular risk^，^ (50–54). However, renal dysfunction may also increase circulating TMAO, indicating that its pathogenic interpretation requires caution (9).

In contrast, SCFAs generally show more consistent cardiovascular protective associations. As fermentation products of dietary fiber, SCFAs contribute to cholesterol metabolism, barrier integrity, and immune regulation (55, 56). Reduced abundance of SCFA-producing bacteria, including *Faecalibacterium* and *Eubacterium rectale*, has been reported in CHD, suggesting early impairment of a protective metabolic network (1, 6, 32, 38). Taken together, the balance between TMAO-related and SCFA-related pathways may be more informative than the absolute level of either metabolite.

### 4.2 Structural dysbiosis and CHD risk

Metagenomic studies have shown that CHD is commonly associated with reduced microbial diversity and compositional deviation. Although findings are not fully consistent across cohorts, several recurrent features have emerged (6, 7, 12). These include an increased *Firmicutes/Bacteroidetes* ratio, enrichment of *Collinsella* associated with inflammatory states and cholesterol dysregulation, and depletion of potentially beneficial taxa such as *Bifidobacterium*, *Prevotella*, and *Rothia* (57–60). By contrast, *Streptococcus* and *other taxa* linked to oral microbial communities appear increased in some CHD cohorts (1, 35, 61, 62). Recent Mendelian randomization analysis further supports a possible causal contribution of specific gut microbial features to CHD risk through metabolic and inflammatory pathways (6).

### 4.3 Immune-inflammatory amplification in the microbiota–gut–vascular axis

A central mechanism connecting gut dysbiosis to CHD is barrier dysfunction and subsequent metabolic endotoxemia. When intestinal integrity is compromised, microbial-associated molecules such as lipopolysaccharide (LPS) can enter the circulation, activate Toll-like receptor 4 (TLR4)-dependent inflammatory pathways, and induce release of IL-6, IL-1β, TNF-α, and related mediators (63). These signals can impair endothelial function and accelerate atherosclerotic progression (55, 64). Clinical observations are broadly consistent with this model, showing enrichment of pro-inflammatory taxa, including *Enterobacteriaceae* and *Streptococcus*, together with depletion of anti-inflammatory bacteria in CHD (65). In this context, likely anti-inflammatory taxa may include *Faecalibacterium*, *Bifidobacterium*, and other SCFA-producing commensals, although their relative contributions may vary across cohorts.

At the immune level, dysbiosis may also influence the T helper 17 (Th17)/ regulatory T (Treg) cells balance, which has been linked to plaque inflammatory activity and differential response to anti-inflammatory therapy (55, 66). Together, these findings suggest that the microbiota–gut–vascular axis functions as an important amplifier of immune-metabolic injury in CHD.

### 4.4 Key microbial metabolites and functional pathways

Beyond compositional changes, microbial function may be more informative than taxonomy alone. For TMAO, inter-individual variation in TMAO-producing capacity may depend more on functional genes such as cutC and cntA than on species names themselves (3, 5, 8, 9).

For SCFAs, butyrate appears particularly important for maintaining intestinal barrier integrity, whereas acetate and propionate are more strongly linked to systemic energy regulation. Proposed mechanisms include histone deacetylase inhibition, promotion of Treg differentiation, and improvement in blood pressure regulation and insulin sensitivity (67–69). These effects collectively support an “SCFAs–metabolism–immunity” protective network, which appears weakened in CHD as SCFAs and their producing bacteria decline (6).

Bile acid metabolism represents another important axis. Secondary bile acids may affect cholesterol handling, energy metabolism, and endothelial inflammation through farnesoid X receptor (FXR)- and takeda G protein-coupled receptor 5(TGR5)-dependent signaling (70, 71).

### 4.5 Clinical multi-omics evidence and host metabolic responses

Multi-omics studies Multi-omics studies link microbial composition, metabolites, and host outcomes (44). CHD signatures include increased TMAO-associated taxa and reduced SCFA-producing bacteria (45, 72). Candida and Streptococcus may contribute to vascular inflammation (73). Clinical indicators of intestinal barrier dysfunction, such as lipopolysaccharide binding protein (LBP) and soluble CD14 (sCD14), may reflect endotoxin exposure, although their specificity remains limited (74–76). Their interpretation may be improved when combined with microbial functional genes and metabolite readouts (77). At the same time, dysbiosis has been linked to activation of multiple inflammatory cascades, including TLR4/NF-*κ*B, the NLRP3 inflammasome, M1 macrophage polarization, and vascular smooth muscle cell activation (8, 10, 77). These pathways likely interact in a cascade-like manner, which may partly explain why some patients respond poorly to single-target anti-inflammatory therapies.

Gut microbiota dysbiosis may also contribute to metabolic disturbance through effects on reverse cholesterol transport, insulin sensitivity, fatty acid oxidation, and oxidative stress (6, 10, 78–81). Reduced butyrate-producing bacteria may favor insulin resistance (79, 80), whereas increased reactive oxygen species (ROS) may further damage endothelial and myocardial cells and destabilize plaques (81). Taken together, current evidence supports a multi-layer model in which gut microbiota dysbiosis contributes to CHD through interconnected pathways involving structural alterations, metabolite imbalance, barrier disruption, immune-inflammatory activation, and host metabolic dysfunction (Figure 1 and Table 1).

## 5 Molecular basis and multi-level mechanisms by which TCM regulates the gut microbiota

**Figure 2:**
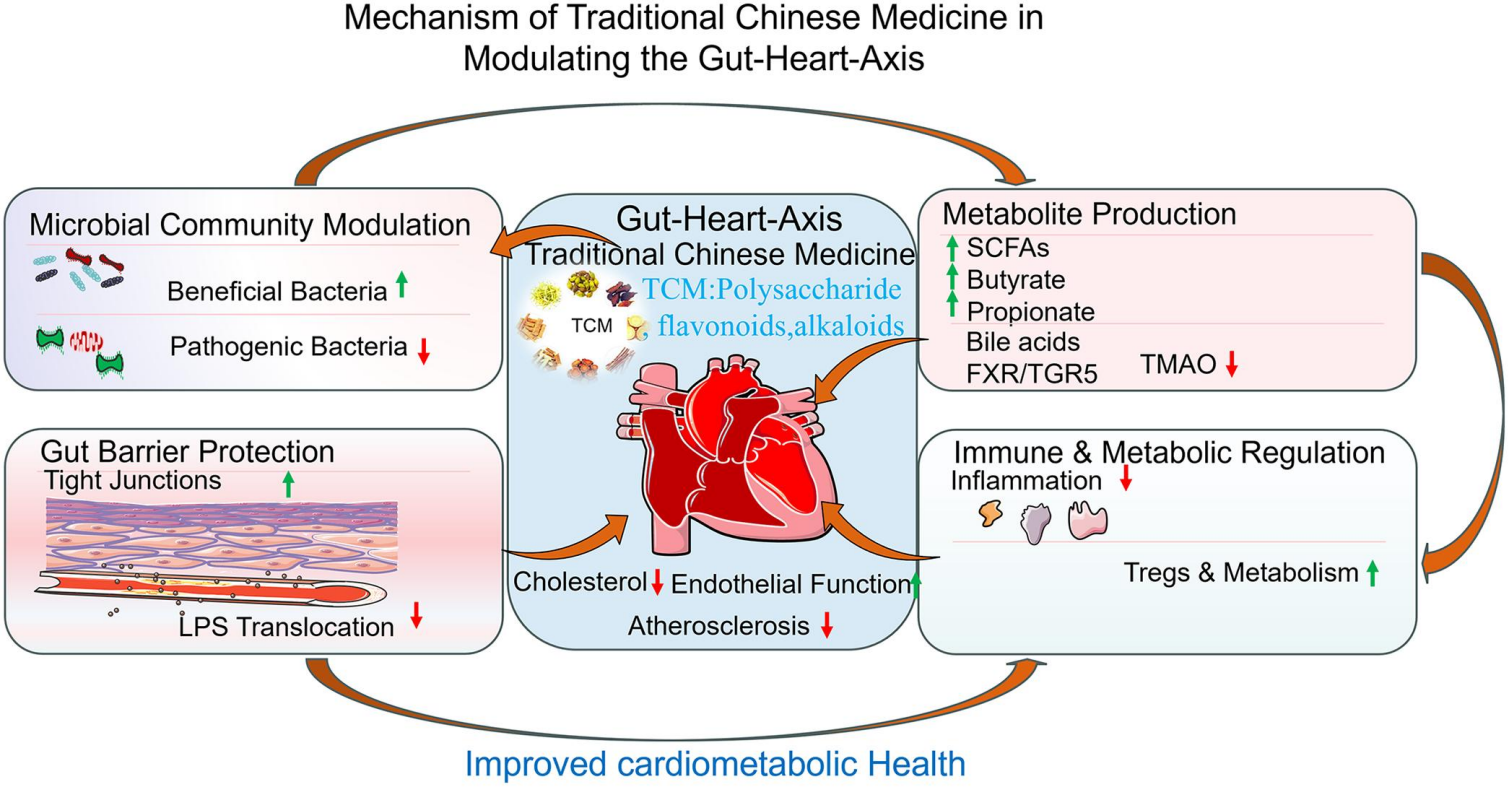
Mechanisms of TCM in modulating the gut–heart axis in CHD. TCM modulates the gut microbiota–host interface by modulating microbial composition, metabolites, intestinal barrier integrity, and immune–metabolic signaling. These effects include enrichment of beneficial taxa reduced endotoxin (LPS) burden, increased SCFA production, altered bile acid pools (FXR/TGR5), and decreased TMAO. Collectively, these changes are associated with improved tight junction function, reduced inflammatory signaling (e.g., TLR4/NF-*κ*B, NLRP3), enhanced regulatory T-cell responses, and improved cardiometabolic homeostasis, contributing to endothelial function and atherosclerosis modulation (67, 82, 83). Major bioactive classes include polysaccharides, saponins, flavonoids, and alkaloids. Arrows indicate regulatory relationships.

With the development of sequencing, metabolomics, and multi-omics integration, the role of TCM in regulating the “microbiota–metabolism–immunity” network has shifted from empirical description to mechanistic interpretation (16–20). Current evidence suggests that TCM may modulate CHD-related pathology through coordinated effects on microbial composition, metabolism, barrier integrity, and host signaling pathways. A schematic overview is presented in Figure 2.

### 5.1 Bidirectional interactions between TCM and the gut microbiota

The gut microbiota is not merely a passive carrier of TCM activity, but an active metabolic partner that contributes to the generation and regulation of its effects (16, 84). Many TCM constituents, particularly polysaccharides, flavonoids, saponins, and phenolic acids, are poorly absorbed in their native form and require microbial biotransformation to yield more bioactive metabolites (16).

On the other hand, TCM itself may remodel gut microbial ecology. Reported effects include suppression of pro-inflammatory taxa such as *Enterobacteriaceae*, enrichment of SCFA-producing bacteria including *Faecalibacterium* and *Eubacterium*, and partial restoration of *α*-diversity and ecological stability (1, 17, 18, 40, 85–87). Through these reciprocal interactions, TCM may act as both a substrate for microbiota-driven metabolism and a regulator of the microbiota–host interface.

### 5.2 Active ingredients and hierarchical regulation of the microbiota–host axis

The complexity of TCM may be advantageous in microecological regulation because different classes of bioactive compounds target distinct microbial and host pathways. Compound formulas, such as Si-Miao-Yong-An Decoction, have been reported to reduce TMAO-related signaling and enhance SCFA-associated protective pathways (88–91). Polysaccharides may primarily support barrier and microbial ecology, flavonoids may contribute to anti-inflammatory and metabolic regulation, and alkaloids may influence microbial functional genes (19, 92).

At the monomer level, Polysaccharides from Astragalus and ginseng may enrich beneficial taxa and enhance SCFA production, supporting their classification as “natural prebiotics” (20, 93–95). Flavonoids (e.g., quercetin, baicalin) and alkaloids (e.g., berberine) show more targeted anti-inflammatory and metabolic effects, including modulation of microbial composition and signaling pathways.

Flavonoids (e.g., quercetin, baicalin) and glycosides (e.g., berberine) may inhibit pro-inflammatory bacteria and activate GPR/Nrf2-related pathways through their metabolites (91, 92). Alkaloids such as berberine and aucubin have been reported to enrich *Akkermansia*, improve metabolic inflammation, and reduce atherosclerosis-associated microbial signatures (96–98).

### 5.3 TCM and intestinal barrier homeostasis

A key mechanism of TCM action is maintenance of intestinal barrier integrity. Herbs such as *Astragalus* and *Codonopsis* have been shown to upregulate tight junction proteins (e.g., ZO-1, occludin), restore mucus layer integrity, reduce intestinal permeability, and lower LPS leakage (19, 36, 45, 55, 98, 99). Barrier protection likely represents a central pathway linking microbiota modulation to reduced systemic inflammation.

### 5.4 Key signaling pathways mediating microbiota–host interactions

TCM-related microbiota regulation converges on several major signaling nodes, including GPR41/43/109A (SCFA signaling), FXR/TGR5 (bile acid metabolism), and inflammatory pathways such as TLR4/NF-*κ*B and NLRP3104-108 (100–102); Additional pathways include Nrf2/HO-1 (oxidative stress) and AMPK/PI3K-Akt-associated tight junction regulation, which supports barrier homeostasis (19, 103, 104).

### 5.5 Synergistic regulation by TCM and probiotics

TCM and probiotics may provide complementary modes of microbiota-targeted intervention. In general, TCM may improve the ecological background of the intestine, whereas probiotics may provide directional reinforcement of specific microbial functions. One major point of convergence is the TMAO axis. Probiotics may further reduce TMA generation from dietary precursors, together forming a potential “precursor inhibition–metabolic redirection–product reduction” strategy (105).

Another important axis is the SCFAs–barrier–immunity pathway. Herbal polysaccharides may function as prebiotics to promote SCFA-associated taxa, increase SCFA production, strengthen barrier integrity, and reduce inflammation (22–24, 106–108). These findings support the concept of combined microecological therapy, although standardization and clinical validation remain limited.

### 5.6 Summary and future directions

Overall, current evidence supports a multi-level role of TCM in modulating the “microbiota–host–metabolism” network in CHD. Future studies should prioritize clinical validation, multi-omics integration, and optimization of dosing and combination strategies. Development of standardized TCM–probiotic formulations may represent a promising direction for microbiota-targeted CHD intervention.

## 6 Research progress of probiotics intervention in CHD

**Figure 3:**
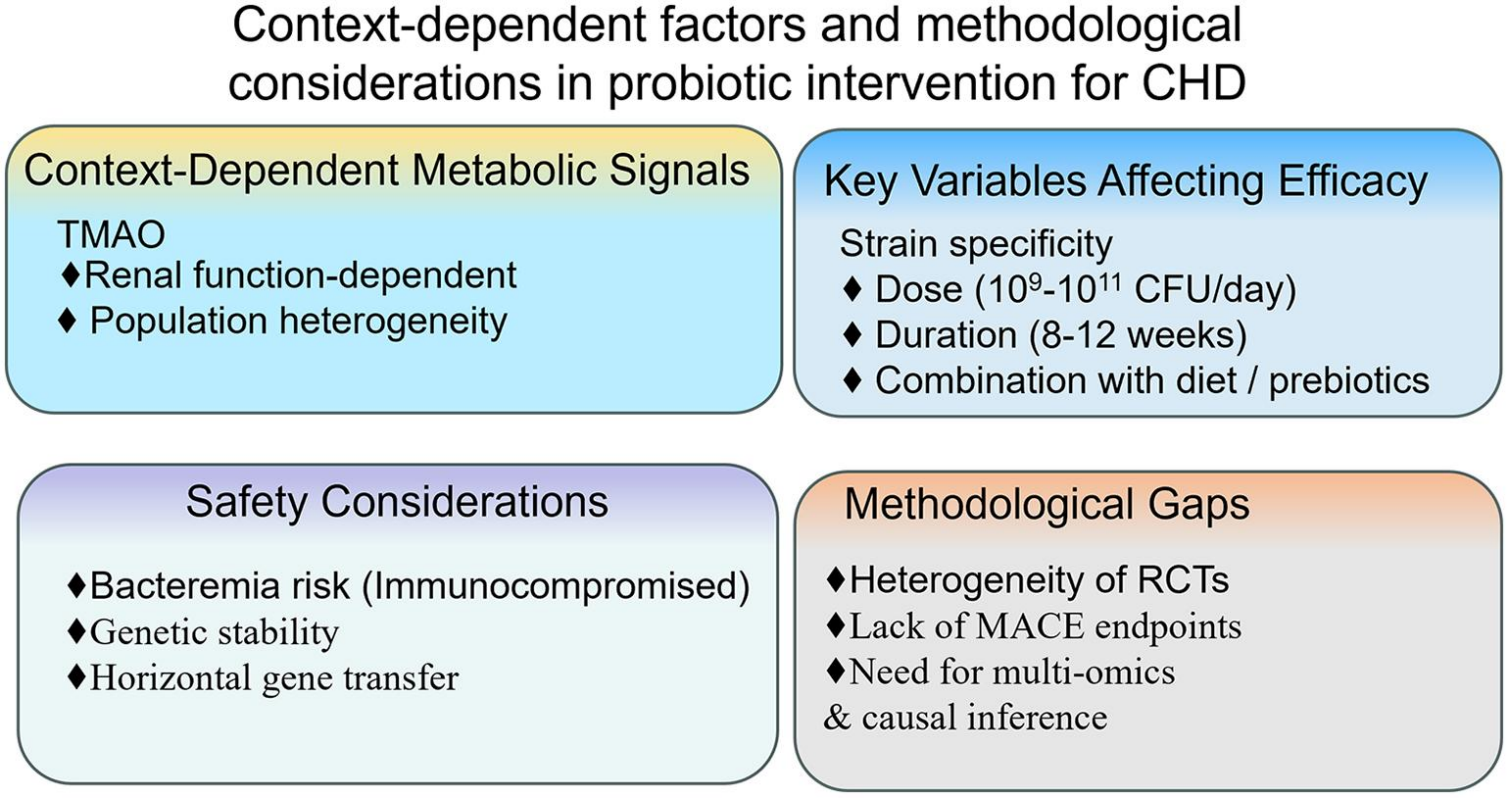
Context-dependent factors and methodological considerations in probiotic intervention for coronary heart disease. This figure summarizes key variables influencing the efficacy and generalizability of probiotic interventions, including strain specificity, dosage, intervention duration, safety considerations, and methodological limitations of current clinical studies. Context-dependent metabolic signals and unresolved challenges that may contribute to heterogeneity across trials are highlighted.

In recent years, probiotics have received increasing attention as a potential microbiota-based strategy for CHD prevention and management. Compared with conventional pharmacological approaches, probiotics act primarily through ecological modulation of the gut–liver–heart axis and may influence host lipid metabolism, bile acid signaling, inflammation, and endothelial function. Emerging evidence suggests that probiotic interventions can generate reproducible signals in improving cardiometabolic risk factors, although their effects on hard clinical outcomes remain uncertain. This section summarizes current progress from four perspectives: clinical evidence, mechanistic pathways, key determinants of efficacy, and safety and translational challenges (Figure 3).

### 6.1 Clinical evidence: promising but heterogeneous

Available randomized controlled trials indicate that the cardiometabolic effects of probiotics are highly strain-dependent. Among the best-studied strains, *Lactobacillus reuteri NCIMB 30242* has shown relatively robust lipid-lowering effects. Clinical studies have shown reductions in LDL-C and total cholesterol, likely related to bile salt hydrolase activity and altered cholesterol handling (25, 29). Subsequent analyses also suggested a possible interaction with vitamin D metabolism (26). Other strains, such as Lactobacillus vaginalis FN3, have shown lipid-lowering potential in experimental models (109), but clinical reproducibility remains limited. *Bifidobacterium species* may improve insulin sensitivity, oxidative stress, and inflammatory status, although direct evidence of stable cardiometabolic benefit in CHD populations is still insufficient (110).

Systematic reviews and meta-analyses generally suggest that probiotics or synbiotics may reduce LDL-C and total cholesterol and improve selected inflammatory markers in individuals with metabolic abnormalities or elevated cardiovascular risk (28–31). However, the overall certainty of evidence is constrained by substantial heterogeneity across studies, including differences in strains, doses, treatment duration, formulations, and background lifestyle factors. Evidence for hard endpoints (e.g., MACE or mortality) remains scarce (5, 111–113). Therefore, current evidence supports probiotics mainly as adjunctive strategies for improving conventional cardiometabolic risk factors rather than as standalone therapies for CHD.

### 6.2 Mechanistic pathways: modulation of the gut–liver–heart axis

**Figure 4:**
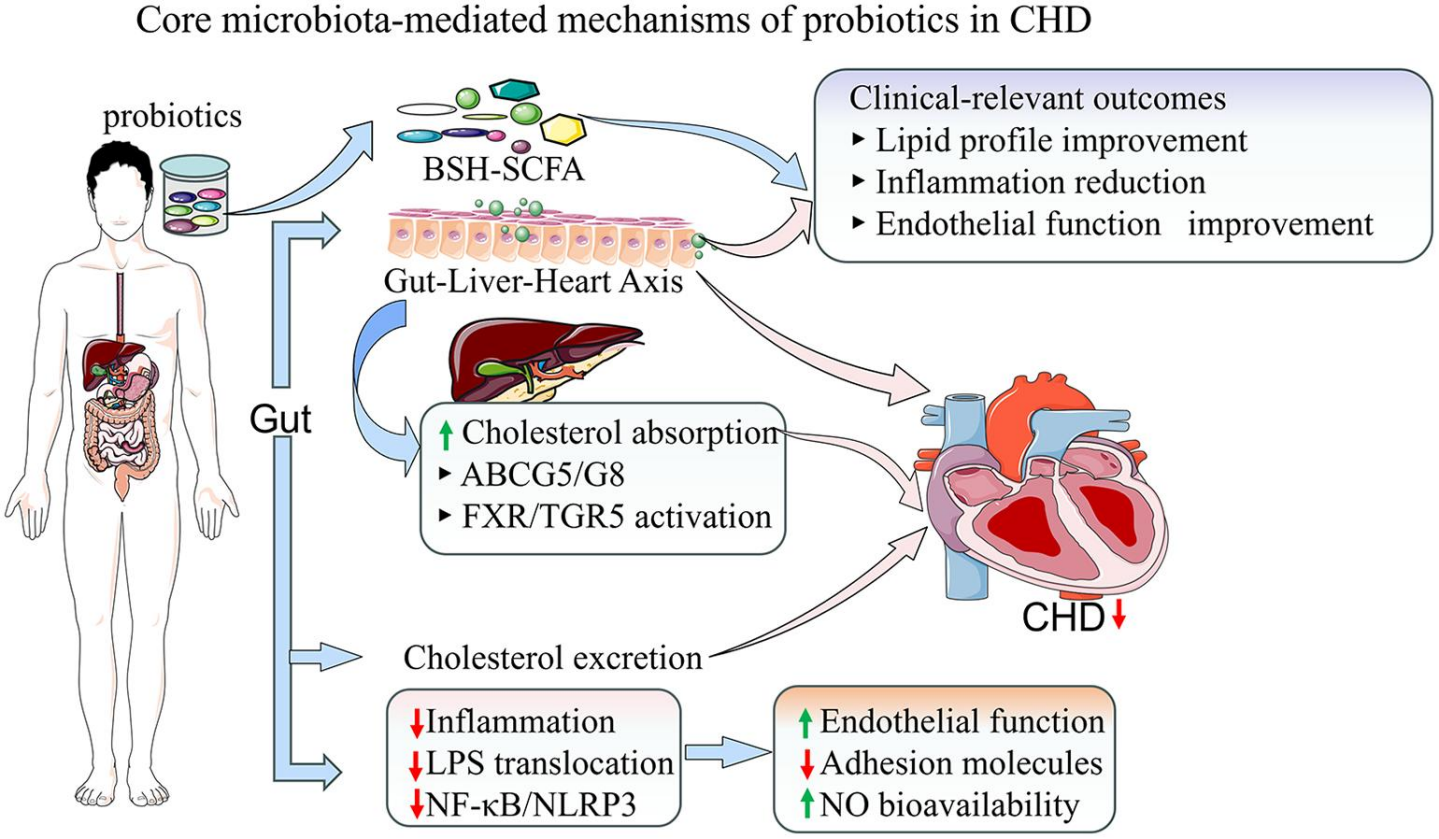
Mechanisms of probiotics in the prevention of CHD via the gut–liver–heart axis. Probiotics influence CHD development primarily through modulation of the gut–liver–heart axis. Probiotic-induced alterations in gut microbiota enhance SCFA production, regulate bile acid metabolism, and reduce endotoxin burden, thereby affecting hepatic signaling pathways such as FXR and TGR5. These metabolic adaptations contribute to decreased formation of pro-atherogenic metabolites, attenuation of inflammatory responses, and improvement of endothelial function. Through coordinated effects on microbial metabolites, immune regulation, and host metabolism, probiotics may mitigate cardiometabolic risk and disease progression.

The strongest mechanistic evidence for probiotic action in CHD relates to bile acid metabolism and cholesterol absorption (Figure 4). First, bile salt hydrolase (BSH) activity in *Lactobacillus* and *Bifidobacterium* promotes bile acid deconjugation, thereby enhancing bile acid excretion and shifting cholesterol metabolism toward bile acid synthesis; this mechanism has been clinically supported for *L. reuteri NCIMB 30242* (25, 29). Second, probiotic-derived metabolites may suppress NPC1L1-mediated cholesterol absorption while increasing ABCG5/G8-mediated efflux, as reported for *L. acidophilus ATCC 4356* and *L. plantarum Lp27* (27, 30, 113, 114). Third, probiotics may alter bile acid composition and thus regulate FXR/TGR5 signaling, linking microbial changes to host lipid metabolism and inflammatory control (27).

Probiotics may also influence key metabolite axes, including SCFAs and TMAO. Briefly, SCFAs contribute to metabolic and immune regulation, whereas TMAO-related effects remain context-dependent and less consistent (5, 115).

Inflammation, oxidative stress, and endothelial dysfunction represent another axis. Probiotics may reduce LPS permeability, suppress NF-*κ*B and NLRP3 signaling, decrease reactive oxygen species production, and improve nitric oxide bioavailability (31, 111).

### 6.3 Determinants of efficacy

Efficacy depends primarily on strain specificity, dose, and duration. Even closely related strains may differ substantially in function (109, 116). Evidence suggests that doses of 10^9^–10^11^ CFU/day administered for at least 8–12 weeks are more likely to yield measurable benefits (29, 116). Combined interventions may improve response consistency. Co-administration with diet or prebiotics may enhance efficacy (30, 31).

In CHD secondary prevention, probiotics are best considered adjunctive to standard therapies rather than replacements.

### 6.4 Safety and translational challenges

Probiotics appear to have a favorable safety profile, but several concerns remain, including bacteremia risk vulnerable populations, genetic instability, and antimicrobial resistance transfer. Methodological limitations affect clinical generalizability. Future trials should standardize strains, doses, and endpoints; include adequate sample size and long-term follow-up for MACE; and incorporate multi-omics approaches (28, 29, 31, 111, 117).

Overall, probiotic interventions show consistent improvements in lipid metabolism and inflammation but lack evidence for hard clinical outcomes. Future progress will depend on combination strategies, microbiome-based stratification, and large-scale mechanistic trials.

## 7 Synergistic mechanisms and comparative advantages of combining TCM and probiotics

With the gut microbiota increasingly recognized as a key contributor to CHD, the multi-level intervention framework of “TCM–probiotics–gut microbiota–host” has shifted from a conceptual hypothesis to a testable strategy. Current evidence suggests that the potential value of this combination lies in functional complementarity between ecological remodeling and targeted metabolic reinforcement. However, it should be noted that direct experimental and clinical evidence supporting such combined interventions specifically in CHD remains limited. Most evidence is derived from related metabolic disorders or indirect mechanistic studies; thus, the proposed synergy should be considered a mechanistically supported but still emerging framework. This section outlines the rationale, mechanistic convergence, and limitations of combined TCM–probiotic intervention in CHD.

### 7.1 Functional complementarity: system remodeling vs. targeted supplementation

If the gut microbiota is viewed as an ecosystem, TCM and probiotics appear to operate at different but complementary levels. TCM, particularly in the form of compound formulas or polysaccharide-rich preparations, primarily reshapes microbial composition, metabolite output, barrier integrity, and inflammatory tone (16, 17, 19–21, 23). In contrast, probiotics provide targeted functional supplementation, includingbile salt hydrolase activity, SCFA production, cholesterol regulation, and inflammatory markers (25, 27–31, 118).

Recent reviews have reinforced this framework, proposing that natural products and probiotics may act on distinct but connected nodes of the microbiota–metabolism–immunity network, thereby producing cross-level complementarity (119). This distinction is illustrated by Si-Miao-Yong-An Decoction, which has been reported to reduce TMAO-related burden and enhance SCFA-associated protective pathways (120). By contrast, strains such as Lactobacillus and Bifidobacterium typically act more selectively on bile acid metabolism, cholesterol absorption, or inflammatory regulation (118). Together, these observations support a working model in which TCM provides “ecological conditioning” whereas probiotics contribute “functional enhancement.” Nevertheless, most supporting evidence remains indirect and derived from separate rather than combined interventions.

### 7.2 Shared mechanistic nodes underlying synergistic effects

The synergistic potential of combined intervention is most evident in several common mechanistic pathways. First, both TCM and probiotics may converge on the TMAO axis. Herbal polyphenols such as resveratrol and alkaloids such as berberine have been reported to reduce TMA/TMAO production by reshaping the microbiota and suppressing microbial functional genes such as cutC and cntA (121–123). probiotics may further reduce precursor availability, forming a coordinated“precursor inhibition–metabolic redirection–product reduction” strategy. However, this coordinated effect has largely been inferred from parallel lines of evidence rather than directly demonstrated in combined intervention models of CHD.

Second, combined intervention may strengthen the SCFAs–barrier–immunit*y* axis. TCM polysaccharides act as prebiotic substrates, enriching such as *Bifidobacterium* and *Lactobacillus*, enhancing barrier integrity, and suppressing low-grade inflammation. When combined with SCFA-associated or bile salt hydrolase-active strains, these effects may amplify barrier-related markers such as ZO-1 and occludin and reinforce GPR- and HDAC-related signaling (22–24). Third, both approaches may converge on inflammation and endothelial protection. TCM formulas such as Qiliqiangxin have shown multi-target anti-inflammatory and remodeling effects in cardiovascular models (21, 22), whereas probiotics may improve nitric oxide bioavailability and reduce LPS permeability, NF-*κ*B/NLRP3 activation, and ROS generation (31, 111). Most evidence originates from non-CHD settingsbut supports a plausible mechanistic basis for synergy.

### 7.3 Multi-omics view: synergy occurs at metabolic crossroads

Multi-omics studies indicate that interactions between TCM and probiotics are concentrated in key metabolic networks rather than microbial taxonomy alone. Three nodes are particularly relevant: the cholesterol–bile acid axis, host–microbiota regulation of TMAO metabolism, and inflammatory signaling networks (4, 36, 70, 102, 120, 124, 125).

Functional outputs such as butyrate may better predict response than taxonomic shifts alone (37, 116), supporting a function-oriented framework for intervention design.

### 7.4 Translational design: from empirical combination to testable strategy

For combined TCM–probiotic intervention to become clinically meaningful, empirical co-administration must be replaced by pathway-based design. A practical strategy may include four elements. First, strain–component matching should target key pathways (e.g., bile acid metabolism, SCFA production, TMAO-related processes) (22, 24, 25, 27–29, 31, 121, 126–129). Second, dose and duration, should be optimized, with evidence suggesting10^9^–10^11^ CFU/day for at least 8–12 weeks is effective (25, 27, 28, 30, 31). Third, outcome assessment should include both clinical endpoints and mechanistic readouts such as TMAO, SCFAs, bile acid profiles, and microbial genes (25, 27, 121, 123, 126). Fourth, population stratification should consider microbiota composition, metabolic status, renal function, and safety factors (28, 29, 111, 117).

### 7.5 Comparative advantages and current boundaries

The advantages of combined TCM–probiotic intervention can be summarized in three aspects. First, a dual-layer mechanism: TCM provides ecological reshaping, while probiotics reinforce specific functions (17, 130, 131). Second, multi-pathway regulation, including bile acid metabolism, TMAO-related signaling, and SCFA-associated pathways (22, 25, 27–29, 31, 121, 126). Third, positioning as an adjunct to standard therapies may enhance feasibility (22, 25, 28, 31, 121, 126).

At the same time, important boundaries remain. There is no unified standard for strain selection, TCM component sourcing, dose, duration, or batch consistency. Long-term ecological stability, resistance gene transmission, opportunistic pathogen competition, and drug–microbiota interactions are also insufficiently characterized (22, 27–29, 31, 132). Therefore, while the mechanistic rationale for synergy is relatively strong, the clinical translation of this combined strategy remains at an early stage.

## 8 Challenges in clinical translation

The “TCM–probiotics–gut microbiota–host” framework progressed toward early translational exploration in CHD. However, major bottlenecks still limit its clinical implementation, including reproducibility, standardization, inter-individual heterogeneity, causal inference, regulatory complexity, and data governance. These challenges are amplified by the complexity of simultaneously modulating microbial ecology and host metabolic networks.

These barriers involve methodological reproducibility, compatibility standardization, inter-individual heterogeneity, causal inference, regulatory compliance, and data governance. Importantly, these challenges are not unique to combined interventions, but are amplified by the complexity of simultaneously modulating both microbial ecology and host metabolic networks.

A fundamental issue is reproducibility. As highlighted by Knight et al., sampling, storage, DNA extraction, sequencing platforms, and bioinformatic pipelines can substantially alter microbiome profiles (133). In the context of combined TCM–probiotic interventions, variability in herbal extraction, batch consistency, strain viability, and colonization dynamics. Thus, adherence to best-practice frameworks such as STORMS and MIxS is essential (133–136).

### 8.1 Standardization and reproducibility

A major translational barrier is the lack of systematic standards for compatibility design. TCM formulas and probiotics act through overlapping pathways such as bile acid metabolism, SCFA production, barrier function, and inflammation (48, 137–139). no unified framework exists for strain–component pairing, dose optimization, or functional persistence TCM extraction processes may alter luminal pH and bile acid composition, thereby influencing probiotic survival and colonization (140), whereas probiotic metabolites, including SCFAs, may in turn affect solubility, transformation, and bioavailability of TCM compounds (120).

Methodological rigor from sample collection to endpoint definition also remains insufficient. Microbiome studies are highly sensitive to sampling site, timing, preservation, extraction methods, sequencing platforms, and batch effects (133, 134, 136, 141). For more complex TCM–probiotic studies, these standards are particularly important because both intervention components and mechanistic endpoints require harmonization. In addition to consistency of herbal fingerprints and strain properties, consensus is needed for endpoint selection, combining conventional clinical outcomes (e.g., LDL-C, hs-CRP, blood pressure, endothelial function) with mechanistic readouts such as TMAO, SCFAs, bile acid profiles, and microbial functional genes (25, 27, 28, 33). Existing ESC recommendations may serve as an appropriate baseline (33).

### 8.2 Individualization and causal verification

Another key challenge is inter-individual heterogeneity. Inter-subject variation remains substantial (37, 142). A more realistic approach is to integrate metagenomics, metabolomics, transcriptomics, and host genetic information to define CHD-related microecological subtypes, such as bile acid axis imbalance, SCFAs–barrier disruption, or TMAO–FMO3 dysregulation (25, 27, 29).

At present, however, most studies remain correlational. Associations between microbiota, metabolites, and CHD establish causality. Mendelian randomization, microbiome-GWAS, and causal graph-based modeling (34).

Clinical translation will require a shift from small, short-term mechanistic studies to randomized and real-world evidence systems. mechanism-enriched multi-center RCTs. This should then be followed by long-term real-world cohorts to assess long-term efficacy and stability.

### 8.3 Evidence hierarchy, regulation, and safety

To enter the evidence-based and regulatory pipeline, TCM, probiotics, and their combinations must be evaluated under internationally accepted frameworks, including SPIRIT, CONSORT, PRISMA 2020, and STROBE (26, 143–145).

For probiotics, clinical translation increasingly overlaps with the regulatory logic of live biotherapeutic products. This introduces additional requirements regarding strain traceability, genotype/phenotype stability, antimicrobial resistance screening, viable count consistency, storage stability, and batch-to-batch reproducibility. When probiotics are combined with TCM, the regulatory complexity further increases.

Safety also requires attention. Microbiota-targeted interventions may alter drug exposure through changes in microbial metabolism, enterohepatic circulation, or host enzyme activity. Future trials should therefore incorporate systematic pharmacovigilance and adhere to ICH E6 (R2)-compatible safety monitoring strategies (146, 147).

### 8.4 Data governance and reusability

In microbiome and multi-omics research, data value depends on reusability and validation. This makes FAIR principles—findability, accessibility, interoperability, and reusability—particularly important (148).

At the methodological level, comparability across studies also depends on open and reproducible analytic workflows. Containerized and traceable analytical frameworks, such as Docker- or Nextflow-based pipelines, may facilitate reproducible multi-cohort integration and accelerate cumulative progress in this field (133, 134).

### 8.5 A realistic translational roadmap

If the “TCM–probiotics–gut microbiota–host” framework is to become a clinically relevant strategy for CHD, translation will likely need to proceed in stages. mechanism validation and safety signals using biomarkers such as TMAO, SCFAs, bile acid profiles, and endothelial function. These data can then support multi-center RCTs in high-risk CHD populations, ideally incorporating both mechanistic endpoints and hard outcomes such as MACE. Finally, large real-world cohorts and post-marketing surveillance systems will be required to monitor long-term efficacy, ecological persistence, safety, and microbiota–drug interactions, while ensuring preparation quality and consistency.

Overall, the combined intervention of TCM and probiotics has entered a stage requiring direct clinical translation efforts. Only under these conditions can this multi-level intervention model evolve into clinically embeddable approach for CHD microecological management.

## 9 Future research

Although substantial progress has been made in understanding the roles of gut microbiota, probiotics, and TCM in CHD, several priorities remain before this field can move from mechanistic insight to clinically actionable intervention. Future work should focus on five directions: AI-assisted systems modeling, precision stratification, interdisciplinary platform construction, translational trial design, and integration of microbiome regulation into secondary prevention frameworks.

### 9.1 AI-assisted modeling of microbiota–host networks

Conventional linear models are insufficient to capture the complex interactions among microbiota, host metabolism, immune signaling, and multi-component interventions. Recent advances in machine learning and graph-based modeling suggest that artificial intelligence may provide useful tools for integrating high-dimensional microbiome, metabolome, and host-response data. Existing models, such as Deep TCM, have already demonstrated the feasibility of simulating multi-target effects of TCM at cellular and molecular levels in complex cardiometabolic settings (140). However, most current models remain centered on the drug–host axis and have not fully incorporated gut microbial ecology.

Future models should therefore integrate microbial composition, microbial function, metabolite flux, and host signaling into a unified and interpretable framework. Such systems should ideally: (i) identify key nodes in microbiota–metabolism–host interactions; (ii) include major gut-derived metabolites such as TMAO, SCFAs, and secondary bile acids as core variables; and (iii) simulate the response to different combinations of TCM components, probiotic strains, or combined interventions. AI-based modeling may serve as a pre-screening tool to prioritize combinations for experimental validation.

### 9.2 Precision intervention under an evidence-based framework

A major reason for the inconsistency of existing probiotic and TCM studies is biological heterogeneity across patients. Response variability is influenced not only by strain, dose, and intervention duration, but also by diet, lifestyle, and baseline microbiome structure. Accordingly, future microbiota-targeted CHD intervention should move from empirical application toward subtype-based precision strategies.

At least three steps appear necessary. First, characteristic microbiome and metabolite signatures of CHD should be identified through metagenomics, metabolomics, and related approaches, including enrichment of TMAO-associated taxa and depletion of SCFA-producing communities (96). Second, dose–response and compatibility databases should be established for probiotic strains, TCM ingredients, and their combinations. Current evidence is still dominated by single-strain, single-dose designs, and robust evidence regarding dose gradients, compatibility ratios, and time-dependent effects is lacking. Third, integration of microbiome stratification with TCM syndrome differentiation may improve interpretability of intervention strategies.

### 9.3 Interdisciplinary research platforms

The complexity of CHD, combined with the multi-component nature of TCM and the ecological variability of probiotics, makes interdisciplinary collaboration essential. Future platforms should include at least three core components.

First, standardized strain libraries and TCM ingredient libraries are needed. Variability in herbal preparation and probiotic strain drift complicates reproducibility (36). A traceable component–strain database with layered quality control would improve consistency. Second, integrated multi-omics platforms combining microbiome, metabolome, and host transcriptome data are required to resolve mechanisms of complex interventions (145). Third, international collaboration networks and multi-center clinical trial systems are necessary to account for geographic variation in diet, genetics, and microbial ecology (146).

### 9.4 From mechanistic studies to clinical implementation

A realistic translational pathway should proceed in stages. Early-phase studies should focus on mechanism-enriched designs with biomarkers such as TMAO, SCFAs, bile acid profiles, endothelial function, and inflammatory signaling. These data can then support dose and regimen optimization, including strain–TCM combinations for different microecological subtypes.

Subsequently, multi-center randomized controlled trials in CHD secondary prevention populations should be conducted, incorporating both mechanistic endpoints and hard clinical outcomes such as MACE. Finally, real-world evidence cohorts and post-marketing surveillance systems will be needed to evaluate durability of effect, long-term compliance, ecological stability, microbiota–drug interactions, and preparation consistency. This staged strategy represents a practical pathway from mechanism to clinical application (33).

## 10 Conclusion

A growing body of evidence supports gut microbiota dysbiosis as a central mechanistic component of CHD progression, linking microbial structure and function to metabolic disturbance, barrier dysfunction, immune-inflammatory activation, and endothelial injury (36, 149). Probiotics provide more directed functional modulation, whereas TCM may exert broader ecological and host-metabolic regulation (33, 150).

However, important barriers remain. Standardized compatibility principles, individualized intervention frameworks, long-term safety assessment, and evidence for hard clinical endpoints are still lacking (13, 32, 33, 46, 96). Thus, current data support serious investigation of this field, but not yet broad clinical recommendation.

Overall, gut microbiota is no longer a passive background factor in CHD, but a potentially actionable node within disease pathogenesis. If current methodological and translational bottlenecks can be addressed, microbiota-targeted strategies—particularly those integrating TCM and probiotics—may become a feasible component of future precision management for CHD.

## References

[1] Jie Z Xia H Zhong SL Feng Q Li S Liang S. The gut microbiome in atherosclerotic cardiovascular disease. Nat Commun. (2017) 8(1):1–12. 10.1038/s41467-017-00900-1 29018189 PMC5635030

[2] Mohsenzadeh A Pourasgar S Mohammadi A Nazari M Nematollahi S Karimi Y. The gut microbiota and cardiovascular disease: exploring the role of microbial dysbiosis and metabolites in pathogenesis and therapeutics. Life Sci. (2025) 381:123981. 10.1016/j.lfs.2025.123981 40987375

[3] Wang Z Klipfell E Bennett BJ Koeth R Levison BS Dugar B. Gut flora metabolism of phosphatidylcholine promotes cardiovascular disease. Nature. (2011) 472(7341):57–63. 10.1038/nature09922 21475195 PMC3086762

[4] Yang Z Liu Y Wang L Lin S Dai X Yan H. Traditional Chinese medicine against COVID-19: role of the gut microbiota. Biomed Pharmacother. (2022) 149:1–37. 10.1016/j.biopha.2022.112787 PMC8901378 35279010

[5] Heianza Y Ma WJ DiDonato JA Sun Q Rimm EB Hu FB. Long-term changes in gut microbial metabolite trimethylamine N-oxide and coronary heart disease risk. J Am Coll Cardiol. (2020) 75(7):763–72. 10.1016/j.jacc.2019.11.060 32081286 PMC8140616

[6] Hu XZ Fu LL Ye B Ao M Yan M Feng HC. Gut microbiota and risk of coronary heart disease: a two-sample Mendelian randomization study. Front Cardiovasc Med. (2024) 11:1273666. 10.3389/fcvm.2024.1273666 38590695 PMC10999620

[7] Bui TVA Hwangbo H Lai Y Hong SB Choi YJ Park HJ. The gut-heart axis: updated review for the roles of microbiome in cardiovascular health. Korean Circ J. (2023) 53(8):499–518. 10.4070/kcj.2023.0048 37525495 PMC10435824

[8] Yang S Li X Yang F Zhao R Pan X Liang J. Gut Microbiota-dependent marker TMAO in promoting cardiovascular disease: inflammation mechanism, clinical prognostic, and potential as a therapeutic target. Front Pharmacol. (2019) 10:1–14. 10.3389/fphar.2019.01360 31803054 PMC6877687

[9] Guasti L Galliazzo S Molaro M Visconti E Pennella B Gaudio GV. TMAO As a biomarker of cardiovascular events: a systematic review and meta-analysis. Intern Emerg Med. (2021) 16(1):201–7. 10.1007/s11739-020-02470-5 32779113

[10] Canyelles M Borrás C Rotllan N Tondo M Escolà-Gil JC Blanco-Vaca F. Gut Microbiota-derived TMAO: a causal factor promoting atherosclerotic cardiovascular disease? Int J Mol Sci. (2023) 24(3):1–17. 10.3390/ijms24031940 PMC9916030 36768264

[11] Wahlström A Sayin SI Marschall HU Bäckhed F. Intestinal crosstalk between bile acids and Microbiota and its impact on host metabolism. Cell Metab. (2016) 24(1):41–50. 10.1016/j.cmet.2016.05.005 27320064

[12] Long D Mao C Zhang X Liu Y Shangguan X Zou M. Coronary heart disease and gut microbiota: a bibliometric and visual analysis from 2002 to 2022. Front Cardiovasc Med. (2022) 9:1–15. 10.3389/fcvm.2022.949859 PMC9493042 36158832

[13] An K Jia Y Xie B Gao J Chen Y Yuan W. Alterations in the gut mycobiome with coronary artery disease severity. EBioMedicine. (2024) 103:1–13. 10.1016/j.ebiom.2024.105137 PMC11087906 38703606

[14] Gregory JC Buffa JA Org E Wang Z Levison BS Zhu W. Transmission of atherosclerosis susceptibility with gut microbial transplantation. J Biol Chem. (2015) 290(9):5647–60. 10.1074/jbc.M114.618249 25550161 PMC4342477

[15] Wu GD Chen J Hoffmann C Bittinger K Chen YY Keilbaugh SA. Linking long-term dietary patterns with gut microbial enterotypes. Science. (2011) 334(6052):105–8. 10.1126/science.1208344 21885731 PMC3368382

[16] Luo T Che Q Guo Z Song T Zhao J Xu D. Modulatory effects of traditional Chinese medicines on gut microbiota and the microbiota-gut-*x* axis. Front Pharmacol. (2024) 15:1–20. 10.3389/fphar.2024.1442854 PMC11497133 39444598

[17] Li D Tang W Wang Y Gao Q Zhang H Zhang Y. An overview of traditional Chinese medicine affecting gut microbiota in obesity. Front Endocrinol (Lausanne). (2023) 14:1–11. 10.3389/fendo.2023.1149751 PMC10016694 36936157

[18] Zhang R Gao X Bai H Ning K. Traditional Chinese medicine and gut microbiome: their respective and concert effects on healthcare. Front Pharmacol. (2020) 11:1–15. 10.3389/fphar.2020.00538 32390855 PMC7188910

[19] Zhu LR Li SS Zheng WQ Ni WJ Cai M Liu HP. Targeted modulation of gut microbiota by traditional Chinese medicine and natural products for liver disease therapy. Front Immunol. (2023) 14:1–14. 10.3389/fimmu.2023.1086078 PMC9933143 36817459

[20] Su M Hu R Tang T Tang W Huang C. Review of the correlation between Chinese medicine and intestinal microbiota on the efficacy of diabetes mellitus. Front Endocrinol (Lausanne). (2022) 13:1–11. 10.3389/fendo.2022.1085092 PMC9905712 36760813

[21] Lu Y Xiang M Xin L Zhang Y Wang Y Shen Z. Qiliqiangxin modulates the gut Microbiota and NLRP3 inflammasome to protect against ventricular remodeling in heart failure. Front Pharmacol. (2022) 13:1–14. 10.3389/fphar.2022.905424 PMC9201726 35721118

[22] Cheang I Yao W Zhou Y Zhu X Ni G Lu X. The traditional Chinese medicine Qiliqiangxin in heart failure with reduced ejection fraction: a randomized, double-blind, placebo-controlled trial. Nat Med. (2024) 30(8):2295–302. 10.1038/s41591-024-03169-2 39095596 PMC11333273

[23] Shan N Wang L Duan C Wu Y Jing Y Fan H. Pretreatment with Astragalus polysaccharide alleviates heat stroke-induced intestinal injury in mice. Front Pharmacol. (2025) 16:1–17. 10.3389/fphar.2025.1612852 PMC12510818 41079737

[24] Huo Z Li J Li X Xiao H Lin Y Ma Y. Functional fractions of Astragalus polysaccharides as a potential prebiotic to alleviate ulcerative colitis. Int J Biol Macromol. (2024) 271(Pt 1):132580. 10.1016/j.ijbiomac.2024.132580 38788871

[25] Jones ML Martoni CJ Prakash S. Cholesterol lowering and inhibition of sterol absorption by Lactobacillus reuteri NCIMB 30242: a randomized controlled trial. Eur J Clin Nutr. (2012) 66(11):1234–41. 10.1038/ejcn.2012.126 22990854

[26] Cusano NE Maalouf NM Wang PY Zhang C Cremers SC Haney EM. Normocalcemic hyperparathyroidism and hypoparathyroidism in two community-based nonreferral populations. J Clin Endocrinol Metab. (2013) 98(7):2734–41. 10.1210/jc.2013-1300 23690312 PMC3701271

[27] Deng C Pan J Zhu H Chen ZY. Effect of gut Microbiota on blood cholesterol: a review on mechanisms. Foods. (2023) 12(23):1–20. 10.3390/foods12234308 PMC10706635 38231771

[28] Chen T Wang J Liu Z Gao F. Effect of supplementation with probiotics or synbiotics on cardiovascular risk factors in patients with metabolic syndrome: a systematic review and meta-analysis of randomized clinical trials. Front Endocrinol (Lausanne). (2023) 14:1–10. 10.3389/fendo.2023.1282699 PMC10801034 38260154

[29] Yao Y Hong Q Ding S Cui J Li W Zhang J. Meta-analysis of the effects of probiotics on hyperlipidemia. Curr Res Food Sci. (2024) 9:1–12. 10.1016/j.crfs.2024.100885 PMC11513789 39469722

[30] Huang Y Zheng Y. The probiotic Lactobacillus acidophilus reduces cholesterol absorption through the down-regulation of Niemann-Pick C1-like 1 in Caco-2 cells. Br J Nutr. (2010) 103(4):473–8. 10.1017/S0007114509991991 19814836

[31] Zhai T Wang P Hu X Zheng L. Probiotics bring new hope for atherosclerosis prevention and treatment. Oxid Med Cell Longev. (2022) 2022:1–13. 10.1155/2022/3900835 PMC9526629 36193065

[32] Yang HT Jiang ZH Yang Y Wu TT Zheng YY Ma YT. Faecalibacterium prausnitzii as a potential Antiatherosclerotic microbe. Cell Commun Signal. (2024) 22(1):1–20. 10.1186/s12964-023-01464-y 38243314 PMC10797727

[33] Tousoulis D Guzik T Padro T Duncker DJ De Luca G Eringa E. Mechanisms, therapeutic implications, and methodological challenges of gut microbiota and cardiovascular diseases: a position paper by the ESC working group on coronary pathophysiology and microcirculation. Cardiovasc Res. (2022) 118(16):3171–82. 10.1093/cvr/cvac057 35420126 PMC11023489

[34] Quan Y Zhang KX Zhang HY. The gut microbiota links disease to human genome evolution. Trends Genet. (2023) 39(6):451–61. 10.1016/j.tig.2023.02.006 36872184

[35] Katsimichas T Theofilis P Tsioufis K Tousoulis D. Gut Microbiota and coronary artery disease: current therapeutic perspectives. Metabolites. (2023) 13(2):1–17. 10.3390/metabo13020256 PMC9963624 36837875

[36] Liu H Zhuang J Tang P Li J Xiong X Deng H. The role of the gut microbiota in coronary heart disease. Curr Atheroscler Rep. (2020) 22(12):77. 10.1007/s11883-020-00892-2 33063240

[37] Wang F Qian F Zhang Q Zhao J Cen J Zhang J. The reduced SCFA-producing gut microbes are involved in the inflammatory activation in Kawasaki disease. Front Immunol. (2023) 14:1–13. 10.3389/fimmu.2023.1124118 PMC10309029 37398673

[38] Zhu Q Gao R Zhang Y Pan D Zhu Y Zhang X. Dysbiosis signatures of gut microbiota in coronary artery disease. Physiol Genomics. (2018) 50(10):893–903. 10.1152/physiolgenomics.00070.2018 30192713

[39] Emoto T Yamashita T Sasaki N Hirota Y Hayashi T So A. Analysis of gut microbiota in coronary artery disease patients: a possible link between gut microbiota and coronary artery disease. J Atheroscler Thromb. (2016) 23(8):908–21. 10.5551/jat.32672 26947598 PMC7399299

[40] Cheng TY Li JX Chen JY Chen PY Ma LR Zhang GL. Gut microbiota: a potential target for traditional Chinese medicine intervention in coronary heart disease. Chin Med. (2021) 16(1):1–20. 10.1186/s13020-021-00516-0 34686199 PMC8540100

[41] Cao L Ni H Gong X Zang Z Chang H. Chinese Herbal medicines for coronary heart disease: clinical evidence, pharmacological mechanisms, and the interaction with gut Microbiota. Drugs. (2024) 84(2):179–202. 10.1007/s40265-024-01994-w 38265546

[42] Wang X Zhang R Zeng N Li H Hua B. Panax notoginseng saponins dually modulates autophagy in gastric precancerous lesions complicated with myocardial ischemia-reperfusion injury model through the PI3K/AKT/mTOR pathway. Biomed Pharmacother. (2024) 178:117268. 10.1016/j.biopha.2024.117268 39116780

[43] Bernini LJ Simao AN Alfieri DF Lozovoy MA Mari NL de Souza CH. Beneficial effects of Bifidobacterium lactis on lipid profile and cytokines in patients with metabolic syndrome: a randomized trial. Effects of probiotics on metabolic syndrome. Nutrition. (2016) 32(6):716–9. 10.1016/j.nut.2015.11.001 27126957

[44] Tenorio-Jimenez C Martinez-Ramirez MJ Del Castillo-Codes I Arraiza-Irigoyen C Tercero-Lozano M Camacho J. Lactobacillus reuteri V3401 reduces inflammatory biomarkers and modifies the gastrointestinal microbiome in adults with metabolic syndrome: the PROSIR study. Nutrients. (2019) 11(8):1–14. 10.3390/nu11081761 PMC6723328 31370223

[45] Cui H Han S Dai Y Xie W Zheng R Sun Y. Gut microbiota and integrative traditional Chinese and western medicine in prevention and treatment of heart failure. Phytomedicine. (2023) 117:154885. 10.1016/j.phymed.2023.154885 37302262

[46] Qu S Deng S Yang T Yang Y Zhang Y Zheng Z. Shengmai Yin alleviated plaque vulnerability and ischemic myocardial damage in diesel exhaust particle-aggravated atherosclerosis with myocardial ischemia. Ecotoxicol Environ Saf. (2022) 234:113379. 10.1016/j.ecoenv.2022.113379 35278994

[47] Zhang Z Yang Z Wang S Wang X Mao J. Natural products and ferroptosis: a novel approach for heart failure management. Phytomedicine. (2025) 142:156783. 10.1016/j.phymed.2025.156783 40286752

[48] Leonov G Livantsova E Varaeva Y Starodubova A. Probiotics and prebiotics in post-myocardial infarction rehabilitation: mechanisms, benefits, and future directions. Curr Nutr Rep. (2025) 14(1):88. 10.1007/s13668-025-00679-4 40580347

[49] Latif F Mubbashir A Khan MS Shaikh Z Memon A Alvares J. Trimethylamine N-oxide in cardiovascular disease: pathophysiology and the potential role of statins. Life Sci. (2025) 361:123304. 10.1016/j.lfs.2024.123304 39672256

[50] Liu G Li J Li Y Hu Y Franke AA Liang L. Gut microbiota-derived metabolites and risk of coronary artery disease: a prospective study among US men and women. Am J Clin Nutr. (2021) 114(1):238–47. 10.1093/ajcn/nqab053 33829245 PMC8277432

[51] Zhou W Cheng Y Zhu P Nasser MI Zhang X Zhao M. Implication of gut microbiota in cardiovascular diseases. Oxid Med Cell Longev. (2020) 2020:1–14. 10.1155/2020/5394096 PMC7533754 33062141

[52] Sanchez-Gimenez R Ahmed-Khodja W Molina Y Peiro OM Bonet G Carrasquer A. Gut Microbiota-derived metabolites and cardiovascular disease risk: a systematic review of prospective cohort studies. Nutrients. (2022) 14(13):1–22. 10.3390/nu14132654 PMC9268449 35807835

[53] Tang WHW Li XS Wu Y Wang Z Khaw KT Wareham NJ. Plasma trimethylamine N-oxide (TMAO) levels predict future risk of coronary artery disease in apparently healthy individuals in the EPIC-Norfolk prospective population study. Am Heart J. (2021) 236:80–6. 10.1016/j.ahj.2021.01.020 33626384 PMC8085024

[54] Fu Q Zhao M Wang D Hu H Guo C Chen W. Coronary plaque characterization assessed by optical coherence tomography and plasma trimethylamine-N-oxide levels in patients with coronary artery disease. Am J Cardiol. (2016) 118(9):1311–5. 10.1016/j.amjcard.2016.07.071 27600460

[55] Kondapalli N Katari V Dalal KK Paruchuri S Thodeti CK. Microbiota in gut-heart axis: metabolites and mechanisms in cardiovascular disease. Compr Physiol. (2025) 15(3):1–22. 10.1002/cph4.70024 PMC12181760 40542540

[56] Luqman A Hassan A Ullah M Naseem S Ullah M Zhang L. Role of the intestinal microbiome and its therapeutic intervention in cardiovascular disorder. Front Immunol. (2024) 15:1321395. 10.3389/fimmu.2024.1321395 38343539 PMC10853344

[57] Turnbaugh PJ Ley RE Mahowald MA Magrini V Mardis ER Gordon JI. An obesity-associated gut microbiome with increased capacity for energy harvest. Nature. (2006) 444(7122):1027–31. 10.1038/nature05414 17183312

[58] Ley RE Turnbaugh PJ Klein S Gordon JI. Microbial ecology: human gut microbes associated with obesity. Nature. (2006) 444(7122):1022–3. 10.1038/4441022a 17183309

[59] Karlsson FH Fak F Nookaew I Tremaroli V Fagerberg B Petranovic D. Symptomatic atherosclerosis is associated with an altered gut metagenome. Nat Commun. (2012) 3:1–8. 10.1038/ncomms2266 PMC3538954 23212374

[60] Astbury S Atallah E Vijay A Aithal GP Grove JI Valdes AM. Lower gut microbiome diversity and higher abundance of proinflammatory genus Collinsella are associated with biopsy-proven nonalcoholic steatohepatitis. Gut Microbes. (2020) 11(3):569–80. 10.1080/19490976.2019.1681861 31696774 PMC7524262

[61] Sayols-Baixeras S Dekkers KF Baldanzi G Jonsson D Hammar U Lin YT. Streptococcus Species abundance in the gut is linked to subclinical coronary atherosclerosis in 8973 participants from the SCAPIS cohort. Circulation. (2023) 148(6):459–72. 10.1161/CIRCULATIONAHA.123.063914 37435755 PMC10399955

[62] Zhou Z Sun L Zhou W Gao W Yuan X Zhou H. Probiotic Bifidobacterium reduces serum TMAO in unstable angina patients via the gut to liver to heart axis. Liver Res. (2025) 9(1):57–65. 10.1016/j.livres.2025.02.001 40206430 PMC11977283

[63] Chen X Zhang H Ren S Ding Y Remex NS Bhuiyan MS. Gut microbiota and microbiota-derived metabolites in cardiovascular diseases. Chin Med J (Engl). (2023) 136(19):2269–84. 10.1097/CM9.0000000000002206 37442759 PMC10538883

[64] Zhang Z Lv T Wang X Wu M Zhang R Yang X. Role of the microbiota-gut-heart axis between bile acids and cardiovascular disease. Biomed Pharmacother. (2024) 174:116567. 10.1016/j.biopha.2024.116567 38583340

[65] Ramirez-Macias I Orenes-Pinero E Camelo-Castillo A Rivera-Caravaca JM Lopez-Garcia C Marin F. Novel insights in the relationship of gut microbiota and coronary artery diseases. Crit Rev Food Sci Nutr. (2022) 62(14):3738–50. 10.1080/10408398.2020.1868397 33399007

[66] Liu H Tian R Wang H Feng S Li H Xiao Y. Gut microbiota from coronary artery disease patients contributes to vascular dysfunction in mice by regulating bile acid metabolism and immune activation. J Transl Med. (2020) 18(1):1–18. 10.1186/s12967-020-02539-x 33036625 PMC7547479

[67] Furusawa Y Obata Y Fukuda S Endo TA Nakato G Takahashi D. Commensal microbe-derived butyrate induces the differentiation of colonic regulatory T cells. Nature. (2013) 504(7480):446–50. 10.1038/nature12721 24226770

[68] Marques FZ Nelson E Chu PY Horlock D Fiedler A Ziemann M. High-Fiber diet and acetate supplementation change the gut microbiota and prevent the development of hypertension and heart failure in hypertensive mice. Circulation. (2017) 135(10):964–77. 10.1161/CIRCULATIONAHA.116.024545 27927713

[69] Pham NHT Joglekar MV Wong WKM Nassif NT Simpson AM Hardikar AA. Short-chain fatty acids and insulin sensitivity: a systematic review and meta-analysis. Nutr Rev. (2024) 82(2):193–209. 10.1093/nutrit/nuad042 37290429 PMC10777678

[70] Sayin SI Wahlstrom A Felin J Jantti S Marschall HU Bamberg K. Gut microbiota regulates bile acid metabolism by reducing the levels of tauro-beta-muricholic acid, a naturally occurring FXR antagonist. Cell Metab. (2013) 17(2):225–35. 10.1016/j.cmet.2013.01.003 23395169

[71] Kida T Tsubosaka Y Hori M Ozaki H Murata T. Bile acid receptor TGR5 agonism induces NO production and reduces monocyte adhesion in vascular endothelial cells. Arterioscler Thromb Vasc Biol. (2013) 33(7):1663–9. 10.1161/ATVBAHA.113.301565 23619297

[72] Prins FM Collij V Groot HE Bjork JR Swarte JC Andreu-Sanchez S. The gut microbiome across the cardiovascular risk spectrum. Eur J Prev Cardiol. (2024) 31(8):935–44. 10.1093/eurjpc/zwad377 38060843

[73] Li Y Liu Y Cui J Zhu M Wang W Chen K. Oral-gut microbial transmission promotes diabetic coronary heart disease. Cardiovasc Diabetol. (2024) 23(1):1–20. 10.1186/s12933-024-02217-y 38581039 PMC10998415

[74] Perez-Diaz-Del-Campo N Castelnuovo G Ribaldone DG Caviglia GP. Fecal and circulating biomarkers for the non-invasive assessment of intestinal permeability. Diagnostics (Basel). (2023) 13(11):1–12. 10.3390/diagnostics13111976 PMC10253128 37296827

[75] Blobaum L Witkowski M Wegner M Lammel S Schencke PA Jakobs K. Intestinal barrier dysfunction and microbial translocation in patients with first-diagnosed atrial fibrillation. Biomedicines. (2023) 11(1):1–13. 10.3390/biomedicines11010176 PMC9856173 36672684

[76] Petrick JL Florio AA Zen J Wang Y Gewirtz AT Pfeiffer RM. Biomarkers of gut barrier dysfunction and risk of hepatocellular carcinoma in the REVEAL-HBV and REVEAL-HCV cohort studies. Int J Cancer. (2023) 153(1):44–53. 10.1002/ijc.34492 36878686 PMC10548479

[77] Witkowski M Weeks TL Hazen SL. Gut microbiota and cardiovascular disease. Circ Res. (2020) 127(4):553–70. 10.1161/CIRCRESAHA.120.316242 32762536 PMC7416843

[78] Ouimet M Barrett TJ Fisher EA. HDL And reverse cholesterol transport. Circ Res. (2019) 124(10):1505–18. 10.1161/CIRCRESAHA.119.312617 31071007 PMC6813799

[79] Qin J Li Y Cai Z Li S Zhu J Zhang F. A metagenome-wide association study of gut microbiota in type 2 diabetes. Nature. (2012) 490(7418):55–60. 10.1038/nature11450 23023125

[80] Cui J Ramesh G Wu M Jensen ET Crago O Bertoni AG. Butyrate-Producing Bacteria and insulin homeostasis: the microbiome and insulin longitudinal evaluation study (MILES). Diabetes. (2022) 71(11):2438–46. 10.2337/db22-0168 35972231 PMC9630078

[81] Xiang M Lu Y Xin L Gao J Shang C Jiang Z. Role of oxidative stress in reperfusion following myocardial ischemia and its treatments. Oxid Med Cell Longev. (2021) 2021:1–23. 10.1155/2021/6614009 PMC8149218 34055195

[82] Zhang K Qin X Qiu J Sun T Qu K Din AU. Desulfovibrio desulfuricans aggravates atherosclerosis by enhancing intestinal permeability and endothelial TLR4/NF-kappaB pathway in Apoe (-/-) mice. Genes Dis. (2023) 10(1):239–53. 10.1016/j.gendis.2021.09.007 37013030 PMC10066333

[83] Yuan X Wang L Bhat OM Lohner H Li PL. Differential effects of short chain fatty acids on endothelial Nlrp3 inflammasome activation and neointima formation: antioxidant action of butyrate. Redox Biol. (2018) 16:21–31. 10.1016/j.redox.2018.02.007 29475132 PMC5842312

[84] Che Q Luo T Shi J He Y Xu DL. Mechanisms by which traditional Chinese medicines influence the intestinal Flora and intestinal barrier. Front Cell Infect Microbiol. (2022) 12:1–10. 10.3389/fcimb.2022.863779 PMC9097517 35573786

[85] Zhu L Zhang D Zhu H Zhu J Weng S Dong L. Berberine treatment increases Akkermansia in the gut and improves high-fat diet-induced atherosclerosis in Apoe(-/-) mice. Atherosclerosis. (2018) 268:117–26. 10.1016/j.atherosclerosis.2017.11.023 29202334

[86] Yang Q Xu Y Shen L Pan Y Huang J Ma Q. Guanxinning tablet attenuates coronary atherosclerosis via regulating the gut Microbiota and their metabolites in Tibetan minipigs induced by a high-fat diet. J Immunol Res. (2022) 2022:1–23. 10.1155/2022/7128230 PMC9352486 35935588

[87] Zhao HR Xian QC Zhang XM Ma XY Wang FQ Wang RS. Jianpi Huayu prescription prevents atherosclerosis by improving inflammation and reshaping the intestinal Microbiota in ApoE(-/-) mice. Cell Biochem Biophys. (2024) 82(3):2297–319. 10.1007/s12013-024-01341-6 39174865 PMC11445337

[88] Kim M Huda MN Bennett BJ. Sequence meets function-microbiota and cardiovascular disease. Cardiovasc Res. (2022) 118(2):399–412. 10.1093/cvr/cvab030 33537709 PMC8803075

[89] Mei Z Chen GC Wang Z Usyk M Yu B Baeza YV. Dietary factors, gut microbiota, and serum trimethylamine-N-oxide associated with cardiovascular disease in the Hispanic community health study/study of Latinos. Am J Clin Nutr. (2021) 113(6):1503–14. 10.1093/ajcn/nqab001 33709132 PMC8168354

[90] Zhang HY Tian JX Lian FM Li M Liu WK Zhen Z. Therapeutic mechanisms of traditional Chinese medicine to improve metabolic diseases via the gut microbiota. Biomed Pharmacother. (2021) 133:1–13. 10.1016/j.biopha.2020.110857 33197760

[91] Zhang M Yang L Zhu M Yang B Yang Y Jia X. Moutan Cortex polysaccharide ameliorates diabetic kidney disease via modulating gut microbiota dynamically in rats. Int J Biol Macromol. (2022) 206:849–60. 10.1016/j.ijbiomac.2022.03.077 35307460

[92] Zhang X Jia L Ma Q Zhang X Chen M Liu F. Astragalus polysaccharide modulates the gut Microbiota and metabolites of patients with type 2 diabetes in an *in vitro* fermentation model. Nutrients. (2024) 16(11):1–18. 10.3390/nu16111698 PMC11174380 38892631

[93] Zhao L Sui M Zhang T Zhang K. The interaction between ginseng and gut microbiota. Front Nutr. (2023) 10:1–14. 10.3389/fnut.2023.1301468 PMC10690783 38045813

[94] Liu W Zhang Y Hu D Huang L Liu X Lu Z. Oral Astragalus polysaccharide alleviates adenine-induced kidney injury by regulating gut microbiota-short-chain fatty acids-kidney G protein-coupled receptors axis. Ren Fail. (2024) 46(2):1–12. 10.1080/0886022X.2024.2429693 PMC11610254 39603250

[95] Feng W Ao H Peng C. Gut microbiota, short-chain fatty acids, and herbal medicines. Front Pharmacol. (2018) 9:1–12. 10.3389/fphar.2018.01354 30532706 PMC6265305

[96] Luo Z Yang L Zhu T Fan F Wang X Liu Y. Aucubin ameliorates atherosclerosis by modulating tryptophan metabolism and inhibiting endothelial-mesenchymal transitions via gut microbiota regulation. Phytomedicine. (2024) 135:156122. 10.1016/j.phymed.2024.156122 39396405

[97] Arenas-Montes J Alcala-Diaz JF Garcia-Fernandez H Gutierrez-Mariscal FM Lopez-Moreno A Luque-Cordoba D. A microbiota pattern associated with cardiovascular events in secondary prevention: the CORDIOPREV study. Eur Heart J. (2025) 46(22):2104–15. 10.1093/eurheartj/ehaf181 40197788

[98] Liang Y Liu M Cheng Y Wang X Wang W. Prevention and treatment of rheumatoid arthritis through traditional Chinese medicine: role of the gut microbiota. Front Immunol. (2023) 14:1–8. 10.3389/fimmu.2023.1233994 PMC10538529 37781405

[99] Li J Xu Y Sun T Zhang X Liang H Lin W. Exploration of the pathogenesis of nephrotic syndrome and traditional Chinese medicine intervention based on gut microbiota. Front Immunol. (2024) 15:1–13. 10.3389/fimmu.2024.1430356 PMC11663840 39717782

[100] Baldrighi M Mallat Z Li X. NLRP3 Inflammasome pathways in atherosclerosis. Atherosclerosis. (2017) 267:127–38. 10.1016/j.atherosclerosis.2017.10.027 29126031

[101] Lu N Cheng W Liu D Liu G Cui C Feng C. NLRP3-Mediated Inflammation in atherosclerosis and associated therapeutics. Front Cell Dev Biol. (2022) 10:1–16. 10.3389/fcell.2022.823387 PMC9045366 35493086

[102] Chiang JYL Ferrell JM. Bile acid receptors FXR and TGR5 signaling in fatty liver diseases and therapy. Am J Physiol Gastrointest Liver Physiol. (2020) 318(3):G554–G73. 10.1152/ajpgi.00223.2019 31984784 PMC7099488

[103] Zhang Q Liu J Duan H Li R Peng W Wu C. Activation of Nrf2/HO-1 signaling: an important molecular mechanism of herbal medicine in the treatment of atherosclerosis via the protection of vascular endothelial cells from oxidative stress. J Adv Res. (2021) 34:43–63. 10.1016/j.jare.2021.06.023 35024180 PMC8655139

[104] Alonso-Pineiro JA Gonzalez-Rovira A Sanchez-Gomar I Moreno JA Duran-Ruiz MC. Nrf2 and heme oxygenase-1 involvement in atherosclerosis related oxidative stress. Antioxidants (Basel). (2021) 10(9):1–21. 10.3390/antiox10091463 PMC8466960 34573095

[105] Ramireddy L Tsen HY Chiang YC Hung CY Wu SR Young SL. Molecular identification and selection of probiotic strains able to reduce the Serum TMAO level in mice challenged with choline. Foods. (2021) 10(12):1–19. 10.3390/foods10122931 PMC8700464 34945482

[106] Chen X Chen C Fu X. Hypoglycemic effect of the polysaccharides from Astragalus membranaceus on type 2 diabetic mice based on the “gut microbiota-mucosal barrier”. Food Funct. (2022) 13(19):10121–33. 10.1039/d2fo02300h 36106494

[107] Li ZY Lin LH Liang HJ Li YQ Zhao FQ Sun TY. Lycium barbarum polysaccharide alleviates DSS-induced chronic ulcerative colitis by restoring intestinal barrier function and modulating gut microbiota. Ann Med. (2023) 55(2):1–17. 10.1080/07853890.2023.2290213 38061697 PMC10836275

[108] Roy S Dhaneshwar S. Role of prebiotics, probiotics, and synbiotics in management of inflammatory bowel disease: current perspectives. World J Gastroenterol. (2023) 29(14):2078–100. 10.3748/wjg.v29.i14.2078 37122604 PMC10130969

[109] Liang X Zhang Z Zhou X Lu Y Li R Yu Z. Probiotics improved hyperlipidemia in mice induced by a high cholesterol diet via downregulating FXR. Food Funct. (2020) 11(11):9903–11. 10.1039/d0fo02255a 33094788

[110] Wlodarska M Willing BP Bravo DM Finlay BB. Phytonutrient diet supplementation promotes beneficial Clostridia species and intestinal mucus secretion resulting in protection against enteric infection. Sci Rep. (2015) 5:1–9. 10.1038/srep09253 PMC4365398 25787310

[111] Zarezadeh M Musazadeh V Ghalichi F Kavyani Z Nasernia R Parang M. Effects of probiotics supplementation on blood pressure: an umbrella meta-analysis of randomized controlled trials. Nutr Metab Cardiovasc Dis. (2023) 33(2):275–86. 10.1016/j.numecd.2022.09.005 36599781

[112] Lv M Shafagh G Yu S. Effect of synbiotics on the cardiovascular risk factors in patients with non-alcoholic fatty liver: a GRADE assessed systematic review and meta-analysis. BMC Gastroenterol. (2025) 25(1):1–20. 10.1186/s12876-025-03789-z 40419987 PMC12107792

[113] Cho YA Kim J. Effect of probiotics on blood lipid concentrations: a meta-analysis of randomized controlled trials. Medicine (Baltimore). (2015) 94(43):1–10. 10.1097/MD.0000000000001714 PMC4985374 26512560

[114] Zhong CY Sun WW Ma Y Zhu H Yang P Wei H. Microbiota prevents cholesterol loss from the body by regulating host gene expression in mice. Sci Rep. (2015) 5:1–12. 10.1038/srep10512 PMC4444975 26015368

[115] Andrikopoulos P Aron-Wisnewsky J Chakaroun R Myridakis A Forslund SK Nielsen T. Evidence of a causal and modifiable relationship between kidney function and circulating trimethylamine N-oxide. Nat Commun. (2023) 14(1):1–18. 10.1038/s41467-023-39824-4 37730687 PMC10511707

[116] Feng H Zhong L Yang X Wu H Sun Q Wei H. Lycium barbarum-probiotic synergy alleviates chemotherapy-induced cancer-related fatigue via gut microbiota-metabolic axis regulation in mice. Front Nutr. (2025) 12:1–12. 10.3389/fnut.2025.1613132 PMC12263555 40672409

[117] Lei Y Xu M Huang N Yuan Z. Meta-analysis of the effect of probiotics or synbiotics on the risk factors in patients with coronary artery disease. Front Cardiovasc Med. (2023) 10:1–12. 10.3389/fcvm.2023.1154888 PMC10436219 37600034

[118] Damluji AA Chung SE Xue QL Hasan RK Walston JD Forman DE. Physical frailty phenotype and the development of geriatric syndromes in older adults with coronary heart disease. Am J Med. (2021) 134(5):662–71. 10.1016/j.amjmed.2020.09.057 33242482 PMC8107119

[119] Qu J Meng F Wang Z Xu W. Unlocking cardioprotective potential of gut microbiome: exploring therapeutic strategies. J Microbiol Biotechnol. (2024) 34(12):2413–24. 10.4014/jmb.2405.05019 39467697 PMC11729380

[120] Pham QH Bui TVA Sim WS Lim KH Law COK Tan W. Daily oral administration of probiotics engineered to constantly secrete short-chain fatty acids effectively prevents myocardial injury from subsequent ischaemic heart disease. Cardiovasc Res. (2024) 120(14):1737–51. 10.1093/cvr/cvae128 38850165 PMC11587561

[121] Li X Su C Jiang Z Yang Y Zhang Y Yang M. Berberine attenuates choline-induced atherosclerosis by inhibiting trimethylamine and trimethylamine-N-oxide production via manipulating the gut microbiome. NPJ Biofilms Microbiomes. (2021) 7(1):1–14. 10.1038/s41522-021-00205-8 33863898 PMC8052457

[122] Li XS Obeid S Klingenberg R Gencer B Mach F Raber L. Gut microbiota-dependent trimethylamine N-oxide in acute coronary syndromes: a prognostic marker for incident cardiovascular events beyond traditional risk factors. Eur Heart J. (2017) 38(11):814–24. 10.1093/eurheartj/ehw582 28077467 PMC5837488

[123] Yang F Gao R Luo X Liu R Xiong D. Berberine influences multiple diseases by modifying gut microbiota. Front Nutr. (2023) 10:1–10. 10.3389/fnut.2023.1187718 PMC10435753 37599699

[124] Lu J Zeng Y Zhong H Guo W Zhang Y Mai W. Dual-Stimuli-Responsive gut microbiota-targeting nitidine chloride-CS/PT-NPs improved metabolic Status in NAFLD. Int J Nanomedicine. (2024) 19:2409–28. 10.2147/IJN.S452194 38476281 PMC10929648

[125] Wen Y Li M Hao Y Peng J Wei X Zhang Z. HDAC/NF-kappaB signaling pathway mediates gut microbiota dysbiosis in rheumatoid arthritis: intervention mechanisms of fengshining decoction. Phytomedicine. (2025) 145:156976. 10.1016/j.phymed.2025.156976 40578039

[126] Chen ML Yi L Zhang Y Zhou X Ran L Yang J. Resveratrol attenuates trimethylamine-N-oxide (TMAO)-induced atherosclerosis by regulating TMAO synthesis and bile acid metabolism via remodeling of the gut Microbiota. mBio. (2016) 7(2):1–14. 10.1128/mBio.02210-15 PMC4817264 27048804

[127] Tawulie D Jin L Shang X Li Y Sun L Xie H. Jiang-Tang-San-Huang pill alleviates type 2 diabetes mellitus through modulating the gut microbiota and bile acids metabolism. Phytomedicine. (2023) 113:154733. 10.1016/j.phymed.2023.154733 36870307

[128] Zhao JA Zheng YZ Wang F Ye JM Guo YF Liang XD. Mulberry water extract alleviates osteoarthritis via Lactobacillus johnsonii-dependent bile acid restoration. Phytomedicine. (2026) 150:157679. 10.1016/j.phymed.2025.157679 41421286

[129] Teichmann J Cockburn DW. *In vitro* fermentation reveals changes in butyrate production dependent on resistant starch source and microbiome composition. Front Microbiol. (2021) 12:1–17. 10.3389/fmicb.2021.640253 PMC8117019 33995299

[130] Yang S Hao S Wang Q Lou Y Jia L Chen D. The interactions between traditional Chinese medicine and gut microbiota: global research status and trends. Front Cell Infect Microbiol. (2022) 12:1–21. 10.3389/fcimb.2022.1005730 PMC9510645 36171760

[131] Yadav M Sehrawat N Sharma AK Kumar S Singh R Kumar A. Synbiotics as potent functional food: recent updates on therapeutic potential and mechanistic insight. J Food Sci Technol. (2024) 61(1):1–15. 10.1007/s13197-022-05621-y 38192708 PMC10771572

[132] Packer M. Qiliqiangxin: a multifaceted holistic treatment for heart failure or a pharmacological probe for the identification of cardioprotective mechanisms? Eur J Heart Fail. (2023) 25(12):2130–43. 10.1002/ejhf.3068 37877337

[133] Knight R Vrbanac A Taylor BC Aksenov A Callewaert C Debelius J. Best practices for analysing microbiomes. Nat Rev Microbiol. (2018) 16(7):410–22. 10.1038/s41579-018-0029-9 29795328

[134] Costea PI Zeller G Sunagawa S Pelletier E Alberti A Levenez F. Towards standards for human fecal sample processing in metagenomic studies. Nat Biotechnol. (2017) 35(11):1069–76. 10.1038/nbt.3960 28967887

[135] Mirzayi C Renson A Genomic Standards C Massive A Quality Control S Zohra F. Reporting guidelines for human microbiome research: the STORMS checklist. Nat Med. (2021) 27(11):1885–92. 10.1038/s41591-021-01552-x 34789871 PMC9105086

[136] Yilmaz P Kottmann R Field D Knight R Cole JR Amaral-Zettler L. Minimum information about a marker gene sequence (MIMARKS) and minimum information about any (x) sequence (MIxS) specifications. Nat Biotechnol. (2011) 29(5):415–20. 10.1038/nbt.1823 21552244 PMC3367316

[137] Wang X Zhou J Jiang T Xu J. Deciphering the therapeutic potential of SheXiangXinTongNing: interplay between gut microbiota and brain metabolomics in a CUMS mice model, with a focus on tryptophan metabolism. Phytomedicine. (2024) 129:155584. 10.1016/j.phymed.2024.155584 38704913

[138] Wang J Zhang H Yuan H Chen S Yu Y Zhang X. Prophylactic supplementation with Lactobacillus Reuteri or its metabolite GABA protects against acute ischemic cardiac injury. Adv Sci (Weinh). (2024) 11(18):1–16. 10.1002/advs.202307233 PMC11095141 38487926

[139] Li B Xu M Wang Y Feng L Xing H Zhang K. Gut microbiota: a new target for traditional Chinese medicine in the treatment of depression. J Ethnopharmacol. (2023) 303:116038. 10.1016/j.jep.2022.116038 36529248

[140] Qian Y Wang X Cai L Han J Huang Z Lou Y. Model informed precision medicine of Chinese herbal medicines formulas-A multi-scale mechanistic intelligent model. J Pharm Anal. (2024) 14(4):1–16. 10.1016/j.jpha.2023.12.004 38694562 PMC11061219

[141] Sinha R Abnet CC White O Knight R Huttenhower C. The microbiome quality control project: baseline study design and future directions. Genome Biol. (2015) 16:1–6. 10.1186/s13059-015-0841-8 26653756 PMC4674991

[142] Wen C Li T Wang B Jin C Li S Li Y. A pectic polysaccharide isolated from Achyranthes bidentata is metabolized by human gut Bacteroides spp. Int J Biol Macromol. (2023) 248:125785. 10.1016/j.ijbiomac.2023.125785 37451376

[143] Vandenbroucke JP von Elm E Altman DG Gotzsche PC Mulrow CD Pocock SJ. Strengthening the reporting of observational studies in epidemiology (STROBE): explanation and elaboration. Epidemiology. (2007) 18(6):805–35. 10.1097/EDE.0b013e3181577511 18049195

[144] Schulz KF Altman DG Moher D Group C. CONSORT 2010 Statement: updated guidelines for reporting parallel group randomised trials. Int J Surg. (2011) 9(8):672–7. 10.1016/j.ijsu.2011.09.004 22019563

[145] Page MJ McKenzie JE Bossuyt PM Boutron I Hoffmann TC Mulrow CD. The PRISMA 2020 statement: an updated guideline for reporting systematic reviews. Rev Esp Cardiol (Engl Ed). (2021) 74(9):790–9. 10.1016/j.rec.2021.07.010 34446261

[146] Bhatt A. The revamped good clinical practice E6(R3) guideline: profound changes in principles and practice!. Perspect Clin Res. (2023) 14(4):167–71. 10.4103/picr.picr_184_23 38025289 PMC10679570

[147] Vischer N Pfeiffer C Joller A Klingmann I Ka A Kpormegbe SK. The good clinical practice guideline and its interpretation—perceptions of clinical trial teams in sub-saharan Africa. Trop Med Int Health. (2016) 21(8):1040–8. 10.1111/tmi.12734 27260671

[148] Wilkinson MD Dumontier M Aalbersberg IJ Appleton G Axton M Baak A. The FAIR guiding principles for scientific data management and stewardship. Sci Data. (2016) 3:1–9. 10.1038/sdata.2016.18 PMC4792175 26978244

[149] Gao H Peng C Wu L Gao S Wang Z Dai L. Yiqi-Huoxue granule promotes angiogenesis of ischemic myocardium through miR-126/PI3K/Akt axis in endothelial cells. Phytomedicine. (2021) 92:153713. 10.1016/j.phymed.2021.153713 34479022

[150] Jia B Zou Y Han X Bae JW Jeon CO. Gut microbiome-mediated mechanisms for reducing cholesterol levels: implications for ameliorating cardiovascular disease. Trends Microbiol. (2023) 31(1):76–91. 10.1016/j.tim.2022.08.003 36008191

